# Genome-Wide Association Study Identifies Genomic Regions for Important Morpho-Agronomic Traits in Mesoamerican Common Bean

**DOI:** 10.3389/fpls.2021.748829

**Published:** 2021-10-07

**Authors:** Jessica Delfini, Vânia Moda-Cirino, José dos Santos Neto, Douglas Mariani Zeffa, Alison Fernando Nogueira, Luriam Aparecida Brandão Ribeiro, Paulo Maurício Ruas, Paul Gepts, Leandro Simões Azeredo Gonçalves

**Affiliations:** ^1^Área de Genética e Melhoramento Vegetal, Instituto de Desenvolvimento Rural do Paraná, Londrina, Brazil; ^2^Departamento de Agronomia, Universidade Estadual de Londrina, Londrina, Brazil; ^3^Departamento de Agronomia, Universidade Estadual de Maringá, Maringá, Brazil; ^4^Departamento de Biologia, Universidade Estadual de Londrina, Londrina, Brazil; ^5^Section of Crop and Ecosystem Sciences, Department of Plant Sciences, University of California, Davis, Davis, CA, United States

**Keywords:** *Phaseolus vulgaris* L., GWAS—genome-wide association study, yield, genetic improvement, favorable alleles

## Abstract

The population growth trend in recent decades has resulted in continuing efforts to guarantee food security in which leguminous plants, such as the common bean (*Phaseolus vulgaris* L.), play a particularly important role as they are relatively cheap and have high nutritional value. To meet this demand for food, the main target for genetic improvement programs is to increase productivity, which is a complex quantitative trait influenced by many component traits. This research aims to identify Quantitative Trait Nucleotides (QTNs) associated with productivity and its components using multi-locus genome-wide association studies. Ten morpho-agronomic traits [plant height (PH), first pod insertion height (FPIH), number of nodules (NN), pod length (PL), total number of pods per plant (NPP), number of locules per pod (LP), number of seeds per pod (SP), total seed weight per plant (TSW), 100-seed weight (W100), and grain yield (YLD)] were evaluated in four environments for 178 Mesoamerican common bean domesticated accessions belonging to the Brazilian Diversity Panel. In order to identify stable QTNs, only those identified by multiple methods (mrMLM, FASTmrMLM, pLARmEB, and ISIS EM-BLASSO) or in multiple environments were selected. Among the identified QTNs, 64 were detected at least thrice by different methods or in different environments, and 39 showed significant phenotypic differences between their corresponding alleles. The alleles that positively increased the corresponding traits, except PH (for which lower values are desired), were considered favorable alleles. The most influenced trait by the accumulation of favorable alleles was PH, showing a 51.7% reduction, while NN, TSW, YLD, FPIH, and NPP increased between 18 and 34%. Identifying QTNs in several environments (four environments and overall adjusted mean) and by multiple methods reinforces the reliability of the associations obtained and the importance of conducting these studies in multiple environments. Using these QTNs through molecular techniques for genetic improvement, such as marker-assisted selection or genomic selection, can be a strategy to increase common bean production.

## Introduction

More than 24 million tons of common beans (*Phaseolus vulgaris* L.) are produced per year worldwide, and the main producing countries are located in Asia and the Americas (Rawal and Navarro, 2019). This crop is mainly grown by small producers, often in low fertility areas with low-level technology, resulting in low mean productivity (Broughton et al., 2003).

Increasing productivity is one of the main objectives of breeding programs. In this context, understanding the genetic constitution related to the productivity and of the production components are the basis for improvement (Kamfwa et al., 2015). In the cultivation of common beans, productivity is related to several morphological, agronomic, and physiological characteristics. The number of pods per plant (NPP), number of seeds per pod (SP), and seed weight are the primary components related to productivity, but other characteristics are also influential, such as growth rate, the capacity of the seeds to absorb photosynthates and plant architecture (Beebe et al., 2013; Resende et al., 2018; Assefa et al., 2019). Physiological and morphological characteristics such as days to flowering and maturity and resistance to pod shattering also significantly impact adaptability, biomass, and productivity (Zhang et al., 2015; Parker et al., 2020a).

Productivity and its components are quantitative traits and are highly influenced by the environment. Thus, understanding the relationship between these traits is very important for directing strategies and efforts in genetic improvement programs (Singh et al., 1991; Assefa et al., 2019). Traditional selection methods in plant breeding require intensive phenotyping fieldwork, with evaluations in several environments and years, resulting in high cost and a time-consuming process (Ikram et al., 2020). The use of molecular markers can increase efficiency and reduce the costs of phenotyping in plant breeding programs. Using molecular tools makes it possible to identify genomic regions related to the productivity and its components, which can be used in marker-assisted selection (Kamfwa et al., 2015). Genome-Wide Association Studies (GWAS) represent a powerful option for the genetic characterization of quantitative traits and have been widely used to analyze agronomic characteristics in plants (Wang et al., 2012; Contreras-Soto et al., 2017; Zhang K. et al., 2017; Cui et al., 2018; Ward et al., 2019; Ikram et al., 2020).

GWAS is a powerful tool enabling the study of different regions of the genome simultaneously using high-resolution mapping. Multiple polymorphisms occurring naturally within a species are identified in germplasm collections with limited genetic structure; genotypes that have traits of interest for breeding programs can be used preferentially to accelerate the application of GWAS studies (Korte and Ashley, 2013). Important genetic factors are identified based on the presence of linkage disequilibrium (LD), while taking into account the potential confounding effect of the population’s structure (Kwak and Gepts, 2009) and kinship relations. Large and highly diverse association panels have unique recombination histories, allowing the detection of small and large genetic effects associated with a particular trait (Oladzad et al., 2019).

Although the statistical power in the detection of Quantitative Trait Nucleotides (QTNs) improves after controlling for the polygenic background of the experimental population under study, most of the small effects associated with complex traits are still not captured by the GWAS single-locus methods (Cui et al., 2018). Single-locus methods perform a one-dimensional scan of the genome; that is, they test one marker at a time, using several rigorous significance test corrections for multiple tests, such as the Bonferroni and False Discovery Rate (FDR) tests. However, these methods can be very conservative in eliminating true QTNs (He et al., 2019b). Multi-locus models are being developed to solve this problem. These models involve a multi-dimensional scanning of the genome, in which the effects of all markers are simultaneously estimated (Cui et al., 2018). The advantage of these models is that it is unnecessary to perform multiple test corrections; therefore, more markers associated with traits of interest are identified (Li et al., 2018).

Several studies seeking to identify allelic variations responsible for traits directly or indirectly related to productivity have already been conducted for common beans (Nemli et al., 2014; Kamfwa et al., 2015; Moghaddam et al., 2016; Soltani et al., 2016; Nascimento et al., 2018; Resende et al., 2018; Lei et al., 2020; Wu et al., 2020). Knowing that abiotic factors, like drought and high temperatures, directly influence production, studies on plant behavior under stress conditions were also conducted (Hoyos-Villegas et al., 2017; Berny Mier y Teran et al., 2018, 2020; Berny Mier Y Teran et al., 2019; Oladzad et al., 2019; Keller et al., 2020; Parker et al., 2020a,b).

Common beans of Mesoamerican origin are the most consumed in Brazil, with a preference for the Carioca and Black commercial classes (Gepts et al., 1988; Burle et al., 2010, 2011; Delfini et al., 2017). Few GWAS have been directed toward diversity panels of plants of Mesoamerican origin and plants adapted to the Brazilian climatic conditions. Studies of genetic variation in accessions adapted to the target habitats are a powerful and effective approach to investigate the genetic architecture of complex traits, and later these natural allelic variations can be directly employed in breeding programs (Nakano and Kobayashi, 2020). Moreover, multi-locus methods were little explored in the cultivation of common beans. In this context, the present study’s objective was to identify genomic regions related to morpho-agronomic traits in Mesoamerican common beans belonging to the Brazilian Diversity Panel (BDP) using the GWAS multi-locus methods.

## Materials and Methods

### Genetic Material, Field Experiments, and Phenotyping

In all, 178 Mesoamerican common bean accessions belonging to the BDP were evaluated (Delfini et al., 2021a). This panel consists of accessions that represent a large part of the variability present in Brazil and are adapted to tropical growing conditions. The phenotyping was conducted at the research stations of the Instituto de Desenvolvimento Rural do Paraná (IDR–Paraná), located in the state of Paraná, Brazil. The experiments were conducted in two seasons: the 2018 rainy season in the cities of Londrina (LDA_A18), Ponta Grossa (PG_A18), and Guarapuava (GUA_18); and the 2018/2019 dry season in Ponta Grossa (PG_S19), totaling four environments. The experiment used an incomplete block design with replications in sets. Five sets with two repetitions were used, and each set covered 50 entries, i.e., 46 accessions and four checks. Each plot consisted of four 2 m long rows, spaced 0.50 m between rows and with a density of 12 plants per linear meter. Management and treatments were conducted according to the technical recommendations for the plant’s cultivation.

Seven plants from the two lateral lines of the plot were used to evaluate traits such as plant height (PH, in cm), first pod insertion height (FPIH, in cm), number of nodules (NN, count variable), pod length (PL, in cm), total number of pods per plant (NPP, count variable), number of locules per pod (LP, count variable), number of seeds per pod (SP, count variable), total seed weight per plant (TSW, in g), and 100-seed weight (W100, in g). Grain yield (YLD, in kg ha^–1^ and 13% moisture) was estimated by harvesting the two central lines.

### Statistical Analysis of Phenotypic Data

An analysis of variance (ANOVA) was conducted using the PROC GLM function in the SAS software (SAS Institute, 2000). The following mathematical model was used:


$$Y_{i\,j\,k\,l}=\mu +A_{i}+S_{j}+A\,S_{i\,j}+R/A\,S_{k\,i\,j}+G/S_{l\,j}+A\,G/S_{m\,l\,j}+e_{i\,j\,k\,l\,m}$$

where is the general mean, *Ai* is the fixed effect of the i-th environment; *S*_*j*_ is the effect of the j-th set; *AS*_*ij*_ is the effect of the interaction between environments and sets; *R/AS_*kij*_* is the effect of the k-th repetition within the interaction between the i-th environment and the j-th set; *G/S_*lj*_* is the random effect of the l-th genotype within the j-th set; *AG/S_*mlj*_* is the effect of the interaction of environments and accessions within the j-th set, and *e*_*ijklm*_ is the experimental error (Hallauer and Miranda Filho, 1988).

The means adjusted for each accession in each of the environments as well as the overall mean of all environments were obtained through the LSmeans option of the GLM procedure. The heritability (*h*^2^) was estimated by the equation: $h^{2}=\sigma_{G}^{2}/\sigma_{P}^{2}$, where genotypic (*σ*^2^*_*G*_*) and phenotypic (*σ*^2^*_*P*_*) variances were estimated by the following equations: $\sigma_{G}^{2}=\left(Q\,M_{G}-Q\,M_{E}\right)$ and $\sigma_{P}^{2}=\left(Q\,M_{G}/r\,a\right)$where *QMG* is the mean square of genotype within sets; *QM*_*E*_ is the mean square of error, *r* is the number of replications, and *a* is the number of environments. The descriptive analysis was determined by the means adjusted for the two replications of each traits in each environment using the PROC UNIVARIATE function in the SAS software. Pearson’s simple linear correlations were calculated and graphically presented using the R software^1^ using the “corrplot” package (Wei and Simko, 2017).

### Genotyping and Genome Wide Association Study

The genotyping-by-sequencing (GBS) technique was used to obtain the SNPs. The methodology used, as well as the results of the population structure and linkage disequilibrium (LD) analyses, are detailed in a previous work (Delfini et al., 2021a). In summary, GBS was conducted using the restriction enzyme *CviAII* (Ariani et al., 2016, 2017) and the data were imputed using Beagle software version 5 (Browning and Browning, 2016). After quality control using VCFtools version 0.1.15 (Danecek et al., 2011), 25,011 SNPs (MAF > 0.05) were used to perform GWAS analyses.

For conducting GWAS, mixed multi-locus models were used with the mrMLM.GUI software version 4.0 0 (Ya-Wen et al., 2019). Four different methods were used: mrMLM (Wang et al., 2016), FASTmrMLM (Tamba and Zhang, 2018), pLARmEB (Zhang J. et al., 2017), and ISIS EM-BLASSO (Tamba et al., 2017). The critical values for significant associations were LOD ≥ 3 for all methods. Population structure and the kinship matrix were included in these models to minimize the identification of false positive associations and increase the statistical power of the analyses. The result of *K = 2* was obtained by the Structure v2.3.4 software (Pritchard et al., 2000) [100,000 burn-in, 100,000 MCMC, and ten repetitions for hypothetical numbers of subpopulations (*K*) between 1 and 10], while the kinship matrix was obtained using the mrMLM.GUI software version 4.0.

The phenotypic values used were the adjusted means for each of the four environments and the overall adjusted mean (LDA_18, PG_18, GUA_18, PG_19, and LSmeans). In order to obtain more accurate results, only QTNs that presented repeatability, that is, detected at least three times by different methods or environments, were considered truly significant and used in the search for favorable alleles and candidate genes.

### Favorable Alleles and Search for Candidate Genes

For each QTN, all accessions were divided into two groups based on the QTN genotype, that is, according to presence or absence of the favorable alleles. A *t*-test was then conducted to test if there was a significant difference in phenotypic mean between the two groups. Only the statistically stable QTNs between the environments, i.e., those that showed significant difference (*P* ≤ 0.05) in the phenotypes in at least three of the five environments (LDA_18, PG_18, GUA_18, PG_19, and LSmeans), were used as favorable alleles. The favorable genotype of each QTN was then selected, i.e., the genotype that causes the desired effect according to each trait, and these effects can be positive or negative in the case of PH. Then, the total number of favorable alleles for each trait was accounted for each accession, and, using a boxplot, visualized if the accumulation of these favorable alleles resulted in phenotypes with more desirable traits.

The identification of candidate genes was conducted at a physical distance of 296 kbp above and below the SNP associated with each of the assessed trait. This distance is the point at which the half decay of the LD, calculated with correction by population structure and relatedness (r^2^_v__s_), occurred (Delfini et al., 2021b). The genes present in the association region with known putative functions according to the GeneOntology (GO)^2^ were identified based on the reference genome annotation of *Phaseolus vulgaris v*.2 published on the Phytozome v10.3 website.^3^

## Results

### Analysis of Variance, Heritability, and Environmental Effect

The analysis of variance showed a significant effect (*P* ≤ 0.01) of accessions and environments for all traits evaluated (Table 1). Significant effects were also observed (*P* ≤ 0.01) for the Genotype x Environment (GE) interactions involving the traits PH, LP, SP, W100, and YLD. The coefficients of variation (CV) varied between 5% (PL) and 26% (TSW). As for heritability estimates (*h*^2^), the TSW, NN, NPP, and FPIH traits presented moderate values, between 0.54 and 0.68, while high values were detected for the other traits, YLD, SP, and LP had *h*^2^*-*values between 0.71 and 0.77, and PH, PL, and W100 had the highest values, 0.88, 0.94, and 0.94, respectively.

**TABLE 1:** Analysis of variance and descriptive statistics for morpho-agronomic traits evaluated in common bean accessions belonging to the Brazilian Diversity Panel (BDP) evaluated in four environments.

		**PH^a^**	**FPIH**	**NN**	**PL**	**NPP**	**LP**	**SP**	**TSW**	**W100**	**YLD**
*F*_*env*_ ^*b*^		827.46***	193.95***	120.58***	369.85***	51.05***	53.95***	88.47***	165.96***	373.06***	627.45***
*F*_*set*_		13.14***	17.57***	9.99***	47.87***	0.85^ns^	34.55***	24.52***	6.82***	1.42^ns^	19.15***
*F*_*env* * *set*_		5.75***	9.78***	11.99***	14.01***	8.84***	10.79***	15.13***	11.58***	8.08***	4.89***
*F*_*rep*(*env* * *set*)_		13.91***	11.04***	7.21***	6.64***	10.25***	10.27***	9.51***	8.69***	5.71***	14.55***
*F*_*treat*(*set*)_		8.17***	3.13***	2.34***	15.78***	2.77***	4.34***	4.06***	2.18***	16^∗∗∗^	3.45***
*F*_*env* * *treat*(*set*)_		1.44***	1.11^ns^	1.07^ns^	1.11^ns^	1.09^ns^	1.23**	1.26**	1.13^ns^	1.61***	1.29***
CV (%)^*c*^		11.77	16.20	10.49	4.79	22.67	6.13	7.38	25.62	7.16	19.10
Heritability (*h*^2^)		0.88	0.68	0.57	0.94	0.64	0.77	0.75	0.54	0.94	0.71
Mean	LDA_18	78.84	19.58	11.67	9.91	17.40	6.79	6.35	16.63	20.95	2368.78
	PG_18	53.46	15.19	10.86	8.98	19.87	6.45	5.90	20.05	24.43	2718.92
	GUA_18	84.26	19.44	12.41	9.91	21.13	6.79	6.35	23.74	23.37	4128.83
	PG_19	72.84	16.61	12.34	9.97	18.80	6.70	6.43	25.18	24.88	3801.88
Minimum	LDA_18	12.81	2.34	1.18	0.81	3.87	0.44	0.52	4.03	2.11	437.90
	PG_18	8.38	1.88	1.00	0.74	3.56	0.46	0.52	4.16	2.73	575.14
	GUA_18	12.55	2.65	1.09	0.77	4.30	0.42	0.48	5.32	2.93	605.74
	PG_19	11.33	4.21	1.32	0.77	4.42	0.49	0.52	6.18	3.31	852.26
Maximum	LDA_18	41.11	12.46	8.61	7.52	8.69	5.55	4.73	8.31	14.69	1110.91
	PG_18	28.84	11.23	6.95	7.13	13.18	5.28	4.75	10.74	17.15	1189.14
	GUA_18	50.48	12.54	8.70	8.10	12.09	5.60	5.01	12.31	16.12	1480.22
	PG_19	41.03	7.13	8.49	8.03	10.45	5.06	4.49	13.63	15.30	1114.61
Skewness	LDA_18	113.26	25.14	14.43	12.47	27.82	7.92	7.48	29.63	26.35	3358.46
	PG_18	81.06	20.18	13.90	11.10	31.37	7.47	7.33	32.59	31.26	4118.57
	GUA_18	122.00	30.44	15.15	12.30	36.43	7.71	7.55	40.73	31.44	5336.69
	PG_19	99.65	28.18	15.67	12.49	36.70	7.68	7.45	43.38	33.48	5691.76
*SD*	LDA_18	–0.072	–0.032	–0.269	0.290	0.363	0.001	–0.286	0.323	–0.116	–0.360
	PG_18	0.035	0.150	–0.486	0.207	0.720	–0.246	0.020	0.242	–0.089	–0.054
	GUA_18	–0.128	0.432	–0.104	0.471	0.567	–0.212	–0.149	0.461	0.071	–0.832
	PG_19	–0.246	–0.147	–0.049	–0.035	0.859	–0.553	–0.776	0.289	–0.082	–0.245
Curtose	LDA_18	0.270	–0.220	–0.070	0.728	–0.348	–0.143	0.045	–0.031	0.022	0.098
	PG_18	0.489	–0.455	1.521	0.101	0.260	–0.383	–0.413	–0.083	–0.465	–0.473
	GUA_18	0.272	0.858	0.300	0.624	0.548	0.064	–0.101	0.324	–0.401	2.382
	PG_19	0.351	–0.244	0.174	0.288	1.252	0.156	0.690	–0.626	–0.013	0.184

*^*a*^PH, plant height (cm); FPIH, first pod insertion height (cm); NN, number of nodules; PL, pod length (cm); NPP, total number of pods per plant; LP, number of locules per pod; SP, number of seeds per pod; TSW, total seed weight per plant (gm); W100, 100-seed weight (gm); YLD, grain yield (kg.ha^–1^).*

^*b*^*F*_*amb*_, *F*_*set*_, *F*_*amb* * *set*_, *F*_*rep*(*amb* * *set*)_, *F*_*trat*(*set*)_, *F*_*amb* * *trat*(*set*)_ represent the values of F for environmental effects, set, interaction between environment and set, repetition within environment and set, treatment within set and interaction between environment, and treatment within set.

^*c*^CV (%) = coefficient of variation. *P < 0.01, **P < 0.001, ***P < 0.0001, and ^*ns*^ not significant.

**FIGURE 1:**
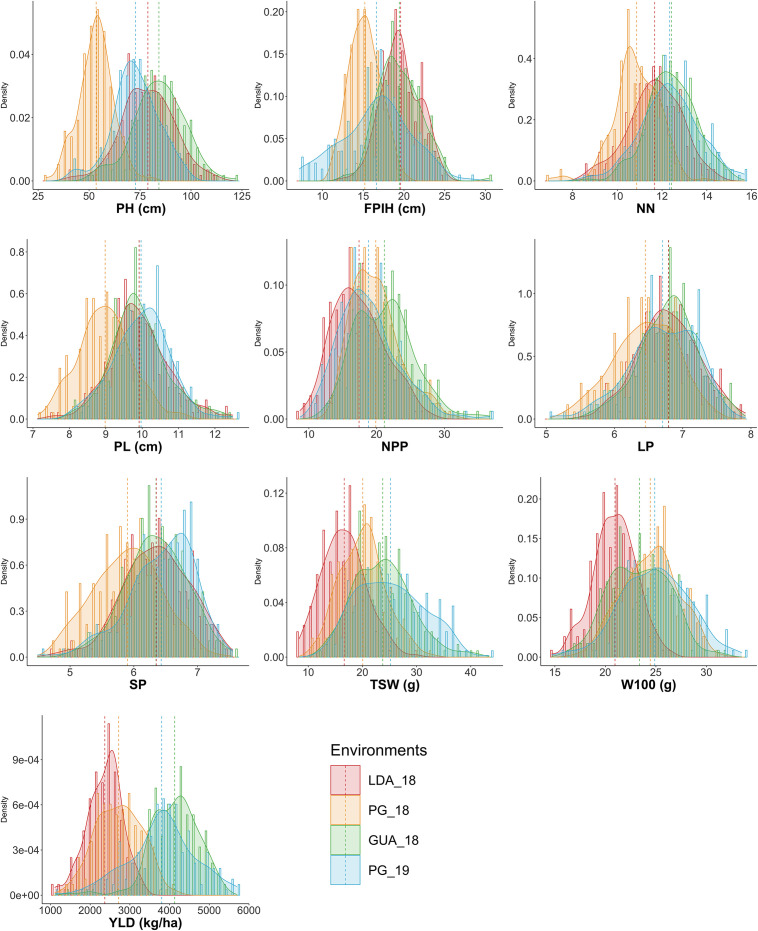
Histogram and density plot of the ten morpho-agronomic traits evaluated in different locations and seasons in common bean accessions belonging to the Brazilian Diversity Panel (BDP). PH, plant height (cm); FPIH, first pod insertion height (cm); NN, number of nodules; PL, pod length (cm); NPP, total number of pods per plant; LP, number of locules per pod; SP, number of seeds per pod; TSW, total seed weight per plant (g); W100, 100-seed weight (g); YLD, grain yield (kg.ha^– 1^). Environments, 2018 rainy season crops: LDA_A18 = Londrina, PG_A18 = Ponta Grossa and GUA_18 = Guarapuava; 2018/2019 dry season crop: PG_S19 = Ponta Grossa.

Comparing the mean performance of the accessions in each of the environments (Figure 1), the GUA_18 environment showed the highest general averages for NPP (21.13) and YLD (4,128.8 kg.ha^–1^), while PG_18 showed the lowest values for traits related to plant morphology: PH (53.46 cm), FPIH (15.19 cm), NN (10.86), and PL (8.98 cm). The LDA_18 environment showed the lowest values for the production components TSW (16.63 cm), W100 (20.95 g), and YLD (2368.78 kg ha^–1^). The traits PL (7.13–12.49 cm), LP (5.06–7.92), SP (4.49–7.55), and W100 (14.69–33.48) did not show significant variations in the minimum, maximum, and mean values for each environment.

### Correlation Between Traits

**FIGURE 2:**
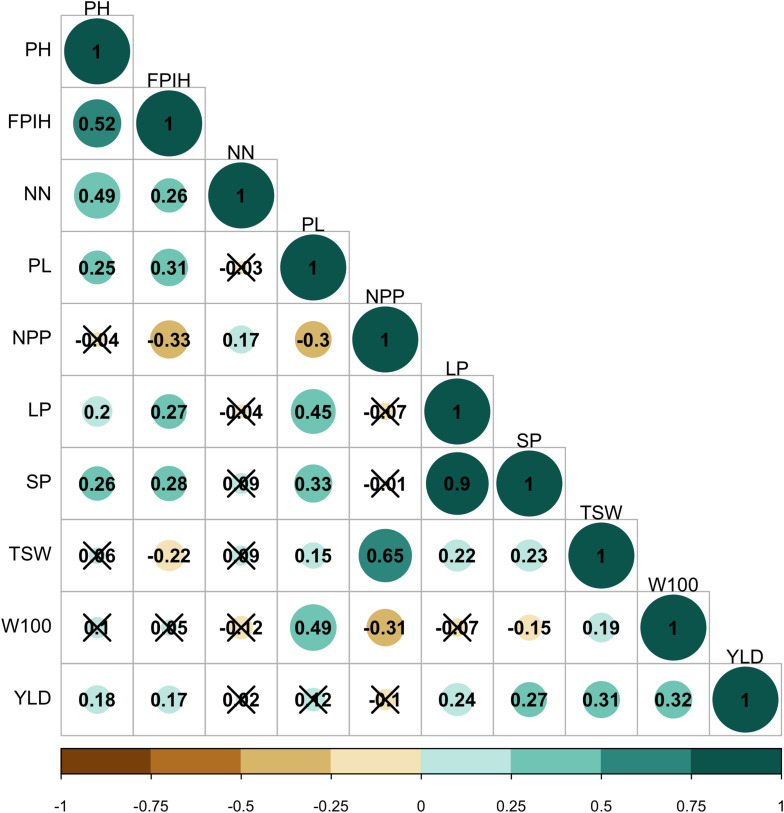
Pearson’s correlation analysis for morpho-agronomic traits of a common bean accession belonging to the Brazilian Diversity Panel (BDP). (X), not significant at 5% probability level. PH, plant height (cm); FPIH, first pod insertion height (cm); NN, number of nodules; PL, pod length (cm); NPP, total number of pods per plant; LP, number of locules per pod; SP, number of seeds per pod; TSW, total seed weight per plant (gm); W100, 100-seed weight (gm); YLD, grain yield (kg.ha^–1^).

Significant and positive correlations (*P* ≤ 0.05) were observed between PH, FPIH, and NN traits. The PL, LP, and SP traits also correlated positively with each other (Figure 2). Positive correlations were also observed between YLD and the primary components TSW and W100 (*r* = 0.31 and 0.32, respectively). In addition, YLD also correlated positively with LP (*r* = 0.24) and SP (*r* = 0.27), while NPP correlated positively with TSW (*r* = 0.65). On the other hand, negative correlations were observed between PPN × FPIH (*r = -*0.33), PPN × PL (*r = −*0.30), PPN × W100 (*r* = *−*0.31), and SP × W100 (*r* = *−*0.15).

### Quantitative Trait Nucleotides Identified by ML-Genome-Wide Association Studies

The four ML-GWAS methods identified 297 QTNs associated with the 10 morpho-agronomic traits evaluated. Among these, 131 QTNs were detected at least twice by two methods and/or two different environments (Supplementary Table 1), while 64 QTNs were detected at least three times by multiple tests and/or multiple environments (Table 2). The highest number of QTNs was observed on the Pv01 and Pv08 chromosomes (nine significant QTNs each), followed by Pv02 and Pv11 (eight QTNs each). Only the 64 QTNs that presented repeatability at least three times were considered reliable and followed in this study.

**TABLE 2:** QTNs associated with morpho-agronomic traits detected at least three times via different methods and in different environments in common bean accessions belonging to the Brazilian Diversity Panel (BDP).

**Trait.**	**SNP**	**Chr**	**Position (bp)**	**QTN Effect^*a*^**	**LOD score^*b*^**	**PVE (%)^*c*^**	**MAF^*d*^**	**Gen**	**Env^*e*^**	**Methods^*f*^**	***T*-test^*g*^**
PH	S01_5305887	1	5,305,887	−8.13∼−6.83	4.78∼6.56	6.55∼9.33	0.063	CC	1	1,2,3	1,5
	S04_2493297	4	2,493,297	2.59∼3.9	3.49∼5.09	2.2∼4.18	0.152	GG	1,5	2,4	1,2,3,5
	S04_373114	4	373,114	−3.34∼−1.73	3.84∼5.66	1.4∼4.6	0.433	GG	3,5	1,4	1,3,5
	S05_39604389	5	39,604,389	1.65∼3.76	3.04∼6.12	2.9∼8.19	0.455	GG	1,2,3,5	1,2,3,4	1,2,3,4,5
	S05_39680093	5	39,680,093	3.16∼4.28	4.86∼7.85	5.51∼10.08	0.352	GG	1	1,2,3	1,2,3,4,5
	S06_25397668	6	25,397,668	2.43∼2.81	3.43∼5.09	2.57∼8.4	0.354	CC	5	1,3,4	1,2,4,5
	S07_17942068	7	17,942,068	5.59∼7.5	5.24∼5.92	2.85∼6.82	0.051	CC	3,4,5	1,4	1,2,3,4,5
	S08_62021856	8	62,021,856	4.08∼8.43	4.52∼6.2	1.84∼9.62	0.062	GG	3,5	3,4	1,2,3,4,5
	S10_43645690	10	43,645,690	2.16∼4.45	4.17∼6.92	3.97∼8.46	0.242	CC	3,5	1,2,3,4	2,3,5
	S10_44036828	10	44,036,828	−2.83∼−1.69	3.04∼4.97	3.89∼10.61	0.393	TT	2	2,3,4	1,2,3,5
	S11_1465100	11	1,465,100	2.69∼3.06	4.78∼5.57	9.01∼11.3	0.318	AA	2	1,2,3	2,3,4,5
FPIH	S01_1292307	1	1,292,307	0.55∼0.59	3.35∼3.43	4.77∼5.28	0.1761	AA	5	1,3,4	4,5
	S01_20991636	1	20,991,636	−0.46∼−0.37	3.33∼3.8	3.62∼5.53	0.4375	CC	5	2,3,4	5
	S06_20814429	6	20814429	−0.61∼−0.49	3.84∼6.86	6.52∼10.05	0.483	AA	5	1,2,4	5
	S09_27171634	9	27,171,634	0.51∼0.78	3.09∼3.63	3.03∼6.93	0.118	CC	2	2,3,4	2
NN	S01_5448199	1	544,8199	0.14∼0.17	3.33∼4.96	2.67∼4.24	0.2584	CC	5	1,2,3	2,4,5
	S02_25464609	2	25,464,609	0.26∼0.36	4∼4.71	4.59∼8.52	0.4148	GG	1	2,3,4	1
	S02_48537121	2	48,537,121	0.17∼0.26	3.03∼3.67	2.4∼5.55	0.2898	CC	2	1,3,4	2,5
	S04_2503984	4	2,503,984	0.13∼0.29	3.67∼5.43	3.03∼7.81	0.3807	CC	2,5	1,2,3	2,3,4,5
	S07_474203	7	474,203	0.12∼0.19	3.12∼4.81	2.07∼5	0.2841	TT	5	1,3,4	1,2,4,5
	S07_495888	7	495,888	−0.35∼0	3.61∼6.22	0∼10.76	0.3523	GG	2	1,2,3,4	2,5
	S08_44008378	8	44,008,378	−0.47∼−0.32	3.84∼5.58	3.42∼7.1	0.0909	CC	2	1,2,3,4	2,3,4,5
	S08_61614494	8	61,614,494	0.15∼0.25	3.93∼7.72	4.14∼11.33	0.4045	TT	5	1,2,3	3,4,5
	S09_5349386	9	5,349,386	0.25∼0.3	3.07∼5.28	2.73∼7.29	0.125	GG	5	1,2,3,4	1,2,3,4,5
	S10_44010107	10	44,010,107	−0.26∼−0.23	3.48∼4.48	4.79∼5.96	0.3708	CC	2	1,2,3	2,5
	S11_8369504	11	8,369,504	−0.19∼−0.14	3.3∼4.87	2.9∼5.43	0.264	AA	5	1,2,3	
PL	S02_1040748	2	1,040,748	−0.26∼−0.19	3.43∼4.19	1.02∼4.9	0.1023	TT	2	1,3,4	1,2,3,5
	S02_47586597	2	47,586,597	−0.33∼−0.22	4.92∼8.61	1.63∼10.93	0.1685	AA	2,3,5	1,2,3,4	1,2,3,5
	S02_49538733	2	49,538,733	0.13∼0.23	3.1∼5.31	2.9∼8.78	0.5	GG	1,2,3	1,2,3	1,2,3
	S07_30515591	7	30,515,591	−0.31∼−0.23	3∼3.65	3.91∼7.18	0.1292	GG	3	1,2,3	1,2,3,5
	S08_1159693	8	1,159,693	−0.23∼−0.19	3.48∼4.51	1.52∼5.23	0.1591	CC	2	1,3,4	1,2
	S08_62432046	8	62,432,046	−0.23∼−0.16	4.01∼4.24	1.08∼5.14	0.1534	TT	2	1,2,3,4	2
	**S08_9375624**	8	9,375,624	−0.54∼−0.41	5.89∼6.76	2.25∼13.21	0.0571	CC	5	2,3,4	1,2,3,4,5
	S11_2437959	11	2,437,959	0.17∼0.18	3.27∼3.39	3.5∼3.89	0.2429	AA	4	1,2,4	2,3,4,5
NPP	S03_12044967	3	12,044,967	−2.28∼−1.62	3.28∼4.46	4.9∼9.69	0.0966	AA	3	1,2,3,4	3,5
	S05_40466290	5	40,466,290	1.04∼1.41	3.12∼5.36	3.96∼7.21	0.2102	TT	3	1,2,3,4	3
	S05_445417	5	445,417	−1.43∼−0.92	3.11∼4.96	2.55∼6.02	0.0562	AA	5	1,2,3	3,5
	S07_38456082	7	38,456,082	−1.56∼−0.48	3.11∼3.85	3.31∼11.71	0.4432	GG	3,4,5	1,2,3,4	3
	S08_2493035	8	249,3035	−2.45∼−2.07	5.78∼6.16	4.1∼11.68	0.1067	CC	4	1,2,4	1,4,5
	**S11_1617681**	11	1,617,681	−0.97∼−0.73	4.94∼7.45	7.5∼13.04	0.4602	AA	5	1,2,3,4	1,2,3,4,5
LP	S01_221817	1	22,1817	0.07∼0.14	3.3∼5.73	1.92∼7.72	0.2921	GG	1	1,2,4	1,5
	S02_41632778	2	41,632,778	0∼0.18	3.03∼3.78	0∼6.56	0.0629	TT	5	1,3,4	2,4,5
	S07_33862545	7	33,862,545	−0.17∼−0.1	3.41∼5.07	3.24 ∼ 9.48	0.1854	AA	3	1,2,3	2,3,4,5
	**S08_9375624**	8	9,375,624	−0.24∼−0.15	3.02∼4.86	4.08∼9.32	0.0514	CC	5	1,2,3,4	2,3,5
	S10_4876917	10	4,876,917	0.12∼0.14	3.41∼3.84	5.38∼6.67	0.2045	AA	3	2,3,4	2,3,5
	S10_4911729	10	4,911,729	0.09∼0.12	3.55∼4.21	4.86∼8.64	0.24	GG	5	1,2,3,4	2,3,5
	S11_29062	11	29,062	−0.13∼−0.07	3.42∼9.02	3.78∼7.81	0.3371	TT	1,5	2,3,4	1,5
	S11_52195944	11	52,195,944	−0.1∼−0.07	3.68∼4.5	4.56∼8.66	0.3616	GG	5	1,2,3	1,5
SP	S01_112055	1	112,055	0.12∼0.2	3.97∼4.81	3.56∼11.2	0.2727	CC	1	2,3,4	1,2,4,5
	S08_10350174	8	10,350,174	−0.37∼−0.13	3.27∼4.79	2.5∼10.42	0.0568	GG	2,5	1,2,3,4	1,2,3,5
TSW	S01_44752890	1	44,752,890	1.98∼2.51	3.53∼4.97	4.17∼8.25	0.1486	CC	4	1,2,3,4	2,4,5
	S02_2242481	2	2,242,481	−1.92∼−1.31	3.4∼6.12	2.89∼6.49	0.2147	TT	4	1,2,4	1,4,5
	S04_41451220	4	41,451,220	−2.03∼−1.53	3.18∼5.58	5.77∼10.15	0.1136	CC	1	2,3,4	1,5
	S10_18589262	10	18,589,262	−3.58∼−1.56	3.48∼4.62	4.49∼8.69	0.0506	GG	3,5	1,2,3,4	3,5
	**S11_1617681**	11	1,617,681	−1.21∼−0.73	3.75∼7.98	5.09∼15.42	0.4571	AA	5	1,2,3,4	2,4,5
	S11_52088416	11	52,088,416	−1.75∼−1.48	4.14∼4.14	5.38∼7.53	0.2247	AA	3	1,2,3	3,5
W100	S03_11484802	3	11,484,802	−0.76∼−0.69	4.42∼5.35	5.42∼6.48	0.4034	CC	3	1,2,3,4	1,2,3,4,5
	S03_4682037	3	4,682,037	0.96∼1.68	3.33∼7.64	4.04∼12.3	0.0686	CC	5	2,3,4	1,4,5
YLD	S01_44911599	1	44,911,599	360.06∼529	3.93∼6.9	6.17∼13.31	0.0966	AA	4	1,2,3,4	1,2,3,4,5
	S01_51067135	1	51,067,135	77.81∼123.27	3.53∼4.87	2.65∼6.45	0.2898	GG	5	1,2,3,4	4,5
	S02_34513049	2	34,513,049	−186.17∼−114.43	3.33∼4.19	1.47∼5.84	0.1875	TT	3	2,3,4	1,2,3
	S03_2802438	3	2,802,438	113.41∼141.54	3.74∼5.64	6.09∼9.49	0.3466	CC	1	2,3,4	1,3,4,5
	S03_49731981	3	49,731,981	−176.06∼−133.48	4.83∼5.89	5.24∼11.65	0.2443	CC	5	1,2,3,4	5
	S07_34450891	7	34,450,891	166.38∼260.05	3.13∼8.89	3.77∼12.15	0.1307	AA	2,4,5	1,2,3,4	1,2,4,5

*PH, plant height (cm); FPIH, first pod insertion height (cm); NN, number of nodules; PL, pod length (cm); NPP, total number of pods per plant; LP, number of locules per pod; SP, number of seeds per pod; TSW, total seed weight per plant (gm); W100, 100-seed weight (gm); YLD, grain yield (kg.ha^–1^).*

*^*a*^Quantitative trait nucleotide effect.*

*^*b*^LOD value, the significant threshold for P-value transformed.*

*^*c*^PVE (%): Phenotypic variation explained.*

*^*d*^Minor allele frequency.*

*^*e*^Environments: 1-LDA, 2-PG, 3-GUA, 4-LSmeans.*

*^*f*^Methods: 1-FASTmrMLM, 2-ISIS EM-BLASSE, 3-mrMLM, 4-pLARmEB.*

*^*g*^QTNs with significant effect on the t-test. Pleiotropic QTNs, related to more than one mineral, are in bold.*

The 64 QTNs identified each explained a low percentage of phenotypic variation (PVE): PH (*n* = 11; PVE = 1.4*-*11.3%), FPIH (*n* = 4; PVE = 3.03-10.05%), NN (*n* = 11; PVE = 3.33.10^–8^-11.33%), PL (*n* = 8; PVE = 1.08-13.21%), NPP (*n* = 6; PVE = 2.55-13.04%), LP (*n* = 8; PVE = 7.03.10^–8^-9.48%), SP (*n = 2;* PVE = 2.5-11.2%), TSW (*n* = 6; PVE = 2.89-15.42%), W100 (*n* = 2; PVE = 4.04-12.3%), and YLD (*n* = 6; PVE = 1.47-13.31%). Two QTNs were considered pleiotropic, since they were identified in more than one trait, i.e., PL-LP (*S08_9375624*) and NPP-TSW (*S11_1617681*) localized on the Pv08 and Pv11 chromosomes, respectively.

In addition to the identification of pleiotropic QTNs, 27 QTNs showed an overlap of their significant genomic regions. On the Pv05, Pv07, and Pv10 chromosomes, QTNs that overlapped for the same trait were observed for PH, NN, and LP, respectively. The PH and NN traits shared the genomic region around the QTNs on three chromosomes: Pv01, Pv04, and Pv10. Other overlaps were observed for SP-LP (Pv01), TSW-YLD (Pv01), W100-NPP (Pv03), LP-YLD (Pv07), PH-LP-PL (Pv08), TSW-LP (Pv11). In addition, the pleiotropic QTN identified for NPP-TSW (Pv11) shared the genomic region with a QTN identified for PH.

The highest number of QTNs was identified in the LSmeans dataset, followed by PG_18, GUA, 18, LDA_18, and PG_19. Among the GWAS multi-locus methods, the ISIS-EM-BLASSO method detected the highest number of SNPs, followed by pLARmEB, mrMLM, and FASTmrMLM. Considering only the 64 reliable QTNs, the environment ranking remained the same, being LSmeans the environment that detected the highest number of QTNs. For the methods, the number of stable QTNs detected was similar among the different methodologies, varying between 53 and 61. Looking at the efficiency of these methods, the number of QTNs considered reliable in relation to the initial number, the FASTmrMLM method stood out from the others (56%), followed by mrMLM (47%), pLARmEB (40%), and ISIS-EM-BLASO (34%).

### Identification of Favorable Allelic Variations and Candidate Genes

**FIGURE 3:**
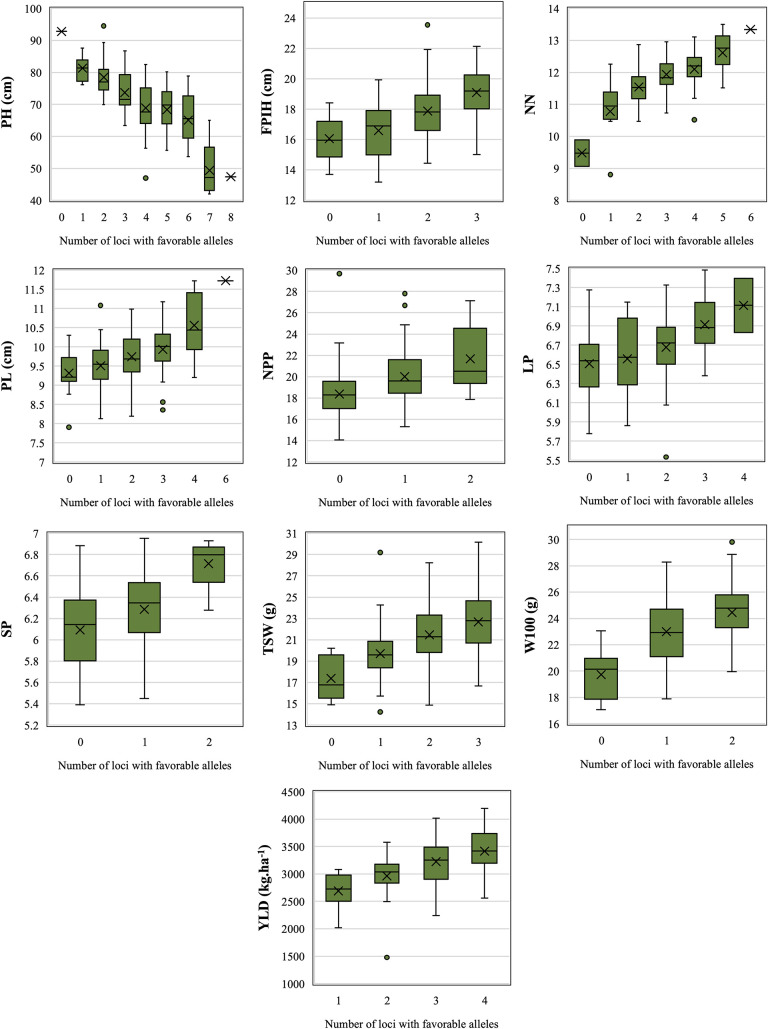
Accumulation of loci with favorable alleles in relation to adjusted means (LSmeans) for morpho-agronomic traits of common beans detected in accessions belonging to the Brazilian Diversity Panel (BDP). PH, plant height (cm); FPIH, first pod insertion height (cm); NN, number of nodules; PL, pod length (cm); NPP, total number of pods per plant; LP, number of locules per pod; SP, number of seeds per pod; TSW, total seed weight per plant (gm); W100, 100-seed weight (gm); YLD, grain yield (kg.ha^–1^).

Among the 64 QTNs considered to be reliable, 39 presented significant results for the *t*-test in at least three environments and were considered stable (Table 2). These stable QTNs were used to identify alleles that were considered to be favorable for the traits PH (*n* = 10), NN (*n* = 6), PL (*n* = 6), NPP (*n* = 2), LP (*n* = 4), SP (*n* = 2), TSW (*n* = 3), W100 (*n* = 2), and YLD (*n* = 4). For FPIH, stable QTNs following the established criteria were not observed, thus, only for this trait, three QTNs that presented significant results through the *t*-test for the overall mean (LSmeans) were used, resulting in a total of 42 stable QTNs (Figure 3).

All QTNs that had a positive effect (increased values) were considered as favorable alleles. The only exception was PH, for which common bean breeding programs search for plants with shorter size, for the purpose of mechanizing the harvest. The accumulation of favorable alleles in the same accession resulted in a gradual increase, or decrease in the case of PH, in all traits (Figure 3). Comparing the mean values between genotypes with zero and those with the maximum number of favorable alleles, PH was the most influenced trait, revealing a difference of 51.7% between the two genotype groups, reducing the values from 92.8 to 48 cm. For those traits where higher values are favorable, the greatest increase was observed for NN (34%, from 9.47 to 12.7), followed by TSW (30%, from 17.4 to 22.7 g), YLD (27%, from 2,688 to 3,419 kg.ha^–1^), W100 (24%, from 19.7 to 24.5 g), FPIH (19%, from 16 to 19 cm), NPP (18%, from 18.4 to 21.7), PL (14%, from 9.3 to 10.6 cm), LP (10%, from 6.5 to 7.14), and SP (10%, from 6.1 to 6.7).

The distance determined using the average LD decay (296 kb) was used to select potential candidate genes at a specific QTN distance. Since the search was conducted in a large genomic region around the QTNs, many genes were identified for the 10 traits evaluated in this study, resulting in 1,528 genes with known putative functions, and of these, 74% were identified more than once in regions of overlap between QTNs. According to GO annotation, the genes were grouped in three functional categories: 55% had a molecular function, 32% had functions related to biological processes, and 13% were cellular components. In the molecular function category, the main functions detected were protein binding, ATP binding, and protein kinase activity; for biological processes, the functions were related to protein phosphorylation, oxidation-reduction processes, and transcription regulation, and for cellular components, the functions were related to the membrane and integral components of the membrane.

## Discussion

Although several studies already identified QTNs associated with morpho-agronomic traits in common beans using GWAS (Nemli et al., 2014; Kamfwa et al., 2015; Moghaddam et al., 2016; Soltani et al., 2016; Nascimento et al., 2018; Resende et al., 2018; Lei et al., 2020; Wu et al., 2020), panels composed exclusively of common beans of Mesoamerican origin and adapted to environmental conditions in Brazil had not been sufficiently explored (Valdisser et al., 2020). Moreover, few studies using the GWAS multi-locus approach have been conducted in common bean. The use of the GWAS multi-locus methods has grown in recent years, becoming one of the main tools to identify molecular markers associated with traits of interest, especially for traits considered complex, i.e., controlled by multiple genes of small effect and highly influenced by the environment (Wen et al., 2018; Zhang et al., 2019; Yang et al., 2020).

A GxE (Genotype by Environment) interaction was observed for most of the morpho-agronomic traits evaluated in the present study, indicating that the accessions’ differential behavior depends on the evaluation environments. The presence of GxE interaction is frequently observed in GWAS studies, where it interferes with the occurrence of the QTN x Environment interaction (Pan et al., 2018; Fattahi and Fakheri, 2019). The estimates of *h*^2^ obtained in the present study were similar to those observed in literature (Rana et al., 2015; Asfaw et al., 2017). The *h*^2^ is the central parameter of any breeding program, used to estimate the response to selection and explain the proportion of phenotypic variation due to genetic variations (Falconer and Mackay, 1996).

Correlations were observed among the traits related to the pods (PL, LP, and SP), the plant architecture traits (PH, FPIH, and NN), and the production components (TSW, W100, and YLD). Similar results were reported in several studies on common beans (Rana et al., 2015; Asfaw et al., 2017; Nadeem et al., 2020). The positive correlations observed between YLD and LP, SP, TSW, and W100 corroborate the possibility of indirect selection of YLD through these traits. However, Asfaw et al. (2017), studying the relationship between morpho-agronomic traits in 202 accessions of Andean and Mesoamerican origin, reported that the correlation between YLD and W100 occurs only in Andean common beans. Furthermore, the same authors recommended the indirect selection of YLD through NPP, independent of the gene pool. Although no positive correlation between YLD and NPP was found in this study, moderate correlations with TSW and SP were observed.

Quantitative genetics assumes that the genetic correlations between traits can be attributed to gene linkage and/or pleiotropy (Saltz et al., 2017). If pleiotropy is the main reason for genetic correlations between two traits, the same QTN can be identified in both traits. However, if gene linkage is the main reason, an overlap of the location between QTNs is expected. Thus, the pleiotropic QTNs identified between the PL-LP and NPP-TSW traits may be considered one of the causes of the correlations observed in these traits. On the other hand, the high number of QTNs identified in overlapping genomic regions indicates that genetic linkage may be the leading cause of the observed genetic correlations in the other assessed traits. Pleiotropy or linkage effects have been reported for many traits such as productivity, biomass, and plant height (Soltani et al., 2016).

Among the ML-GWAS methods used, the ISIS-EM-BLASSO detected the highest number of SNPs. However, it was the least efficient based on the number of verified QTNs. On the other hand, the FASTmrMLM method, while it detected the lowest number of QTNs detected, was considered the most efficient of the methods evaluated. Several studies comparing the ISIS-EM-BLASSO, pLARmEB, mrMLM, and FASTmrMLM methods have already been performed in many crops and the results are similar to those observed in the present study (Ma et al., 2018; Misra et al., 2018; Zhang et al., 2018; Fang et al., 2020). Although the ML-GWAS methods have similar approaches, the differential identification of QTNs is related to different screening and estimation models of each method (Zhang et al., 2018). From the present study results, FASTmrMLM can be considered the most reliable method, as it presented a low rate of false-positive associations. The FASTmrMLM method results from an improvement of the mrMLM method, which is a faster, more reliable, with higher statistical power, higher estimation accuracy, and low false-positive rate (Tamba and Zhang, 2018).

Most of the QTNs identified in this study were observed in only one environment, indicating the presence of frequent QTN x Environment interactions. Several studies have reported previously the presence of these morpho-agronomic traits interaction in common beans, suggesting that the gene expression of these QTNs is influenced by the evaluation environment (MacQueen et al., 2020; Wu et al., 2020). The presence of the QTN x Environment interaction is considered one of the main challenges in selecting QTNs in breeding programs, as these QTNs are more prone to environmental effects. On the other hand, the stable QTNs provided a remarkable demonstration of gradual improvement of all the traits evaluated in this study through the accumulation of favorable alleles.

Significant increases in productivity (YLD) and its primary components (NPP, SP, TSW, W100) were observed, and alleles that caused a significant PH reduction were identified. The PH is an essential factor in the formation of production components, and at the same time, it promotes or inhibits other components, affecting mainly the resistance to lodging, NN and NPP (Fang et al., 2020). Small-sized plants are associated with a determinate growth habit, less susceptibility to lodging, and shorter cycles (Chang et al., 2018). Over the years, with the technification of agriculture, common bean breeding programs have sought to develop plants with these traits, as they facilitate management and mechanized harvesting, reducing harvest losses and susceptibility to some diseases, allowing an increase in the number of crops per year due to a reduction in the cycle (Teixeira et al., 1999).

Most QTNs identified in this study had a small effect, confirming the complex and quantitative nature of the main morpho-agronomic traits in common beans (Gupta et al., 2020; MacQueen et al., 2020). As most of the traits studied are controlled by polygenes, the effect of each locus individually is relatively small. Nevertheless, it is vital to identify small-effect loci that cumulatively can explain the variation in a trait (Nakano and Kobayashi, 2020). The selection of higher-effect QTNs is preferable for the selection assisted by molecular markers (SAM) (Nadeem et al., 2018; Oladosu et al., 2019). However, the use of small-effect QTNs associated with the traits of interest is considered an important strategy in approaches to genomic selection (GS) since only these QTNs can replace the need for high-density genotyping by random SNPs and thus reduce genotyping costs. Moreover, models of GS using only SNPs known to be associated with the traits of interest showed greater accuracy of prediction since they showed lower background noise in constructing these models (He et al., 2019a; Ali et al., 2020).

Among the candidate gene models identified in this study, nine (*Phvul.001G189200*, *Phvul.001G192200*, *Phvul.003G039900*, *Phvul.006G098300, Phvul.007G246700*, *Phvul.008G013300*, *Phvul.008G268700*, *Phvul.008G277352*, and *Phvul.011G020500*) were previously identified in other studies of GWAS for morpho-agronomic traits in common beans (Cichy et al., 2015; Moghaddam et al., 2016; Soltani et al., 2017; Tock et al., 2017; MacQueen et al., 2020). The candidate gene model *Phvul.003G039900*, associated with W100 in the present study, was also identified for seed weight by MacQueen et al. (2020). The same authors observed an association between the gene model *Phvul.006G098300* and PH, whereas this gene was associated with FPIH in the present study. The gene model *Phvul.003G039900* has a putative methyltransferase activity function and the gene model *Phvul.006G098300* is related to transferase activity and transferring acyl groups other than amino-acyl groups.

The candidate gene model *Phvul.008G013300*, related to PL in this study, was also associated with the weight of seeds as reported by Moghaddam et al. (2016) and has a serine-type endopeptidase activity and proteolysis functions. The candidate gene model *Phvul.011G020500* was associated with PH, NPP, and TSW, while this same gene model was associated with the aerial part’s biomass trait in the observations of Soltani et al. (2017). Several functions were reported for this gene, such as DNA-binding transcription factor activity, transcription regulator complex, regulation of transcription and cell cycle.

Considering that the genomic regions around the significant QTNs for the different traits assessed in this study overlapped one another, 74% of the identified genes were also detected for more than one trait. Due to the strong LD observed in common beans, it is difficult to assign a gene precisely to a trait, especially when it comes to polygenic traits (Ikram et al., 2020). The genes located in the genomic regions around the identified QTNs may serve as promising targets for studying molecular mechanisms responsible for morpho-agronomic traits in common beans.

In this study, 64 QTNs were identified for ten morpho-agronomic traits in common beans. Thirty-nine of them were identified as favorable alleles that can significantly increase trait expression and potentially yield and its components in the cultivation of common beans through allele pyramiding. The results reinforce the importance of conducting phenotyping of individuals in multiple environments, using multiple detection methods to increase the reliability of QTNs obtained in GWAS studies. The QTNs identified proved adequate for implementation in common bean breeding programs, mainly for improving Mesoamerican common beans from the Black and Carioca commercial classes, which are the primary targets in Brazil.

## Data Availability Statement

The raw data supporting the conclusions of this article will be made available by the authors, without undue reservation.

## Author Contribuitions

JD, VM-C, and LG conceived and designed the study. JD collected plant material, extracted DNA, and performed the genotyping. JD, JS, AN, LR, and DZ performed the phenotyping. JD and LG performed bioinformatics and statistical analyses. JD and DZ drafted the manuscript. JD, VM-C, JS, DZ, PR, PG, and LG edited and revised the final manuscript. All authors read and approved the final manuscript.

## Conflict of Interest

The authors declare that the research was conducted in the absence of any commercial or financial relationships that could be construed as a potential conflict of interest.

## Publisher’s Note

All claims expressed in this article are solely those of the authors and do not necessarily represent those of their affiliated organizations, or those of the publisher, the editors and the reviewers. Any product that may be evaluated in this article, or claim that may be made by its manufacturer, is not guaranteed or endorsed by the publisher.

## Footnotes

1
https://www.r-project.org/

2
http://www.geneontology.org/

3
http://phytozome.net

## References

[B1] Ali M. Zhang Y. Rasheed A. Wang J. Zhang L. (2020). Genomic prediction for grain yield and yield-related traits in chinese winter wheat. *Int. J. Mol. Sci.* 21:1342. 10.3390/ijms21041342 32079240 PMC7073225

[B2] Ariani A. Berny Mier y Teran J. C. Gepts P. (2016). Genome-wide identification of SNPs and copy number variation in common bean (*Phaseolus vulgaris L*.) using genotyping-by-sequencing (GBS). *Mol. Breed.* 36:87. 10.1007/s11032-016-0512-9

[B3] Ariani A. Berny Mier y Teran J. C. Gepts P. (2017). Spatial and temporal scales of range expansion in wild *Phaseolus vulgaris*. *Mol. Biol. Evol.* 35 119–131. 10.1093/molbev/msx273 29069389 PMC5850745

[B4] Asfaw A. Ambachew D. Shah T. Blair M. W. (2017). Trait associations in diversity panels of the two common bean (*Phaseolus vulgaris l*.) gene pools grown under well-watered and water-stress conditions. *Front. Plant Sci.* 8:733. 10.3389/fpls.2017.00733 28536592 PMC5422517

[B5] Assefa T. Assibi Mahama A. Brown A. V. Cannon E. K. S. Rubyogo J. C. Rao I. M. (2019). A review of breeding objectives, genomic resources, and marker-assisted methods in common bean (*Phaseolus vulgaris L*.). *Mol. Breed.* 39:20. 10.1007/s11032-018-0920-0

[B6] Beebe S. E. Rao I. M. Blair M. W. Acosta-Gallegos J. A. (2013). Phenotyping common beans for adaptation to drought. *Front. Physiol.* 4:35. 10.3389/fphys.2013.00035 23507928 PMC3589705

[B7] Berny Mier Y Teran J. C. Konzen E. R. Palkovic A. Tsai S. M. Rao I. M. Beebe S. (2019). Effect of drought stress on the genetic architecture of photosynthate allocation and remobilization in pods of common bean (*Phaseolus vulgaris L*.), a key species for food security. *BMC Plant Biol.* 19:171. 10.1186/s12870-019-1774-2 31039735 PMC6492436

[B8] Berny Mier y Teran J. C. Konzen E. R. Medina V. Palkovic A. Ariani A. Tsai S. M. (2018). Root and shoot variation in relation to potential intermittent drought adaptation of Mesoamerican wild common bean (*Phaseolus vulgaris L*.). *Ann. Bot.* 124 917–932. 10.1093/aob/mcy221 30596881 PMC6881220

[B9] Berny Mier y Teran J. C. Konzen E. R. Palkovic A. Tsai S. M. Gepts P. (2020). Exploration of the yield potential of mesoamerican wild common beans from contrasting eco-geographic regions by nested recombinant inbred populations. *Front. Plant Sci.* 11:346. 10.3389/fpls.2020.00346 32308660 PMC7145959

[B10] Broughton W. J. Hernandez G. Blair M. Beebe S. Gepts P. Vanderleyden J. (2003). Beans (*Phaseolus spp*.)–model food legumes. *Plant Soil* 252 55–128. 10.1023/A:1024146710611

[B11] Browning B. L. Browning S. R. (2016). Genotype imputation with millions of reference samples. *Am. J. Hum. Genet.* 98 116–126. 10.1016/j.ajhg.2015.11.020 26748515 PMC4716681

[B12] Burle M. L. Fonseca J. R. del Peloso M. J. Melo L. C. Temple S. R. Gepts P. (2011). Integrating phenotypic evaluations with a molecular diversity assessment of a brazilian collection of common bean landraces. *Crop Sci.* 51 2668–2680. 10.2135/cropsci2010.12.0710

[B13] Burle M. L. Fonseca J. R. Kami J. A. Gepts P. (2010). Microsatellite diversity and genetic structure among common bean (*Phaseolus vulgaris L*.) landraces in Brazil, a secondary center of diversity. *Theor. Appl. Genet.* 121 801–813. 10.1007/s00122-010-1350-5 20502861 PMC2940433

[B14] Chang F. Guo C. Sun F. Zhang J. Wang Z. Kong J. (2018). Genome-wide association studies for dynamic plant height and number of nodes on the main stem in summer sowing soybeans. *Front. Plant Sci.* 9:1184. 10.3389/fpls.2018.01184 30177936 PMC6110304

[B15] Cichy K. A. Porch T. G. Beaver J. S. Cregan P. Fourie D. Glahn R. P. (2015). A Phaseolus vulgaris diversity panel for Andean bean improvement. *Crop Sci.* 55 2149–2160. 10.2135/cropsci2014.09.0653

[B16] Contreras-Soto R. I. Mora F. De Oliveira M. A. R. Higashi W. Scapim C. A. Schuster I. (2017). A genome-wide association study for agronomic traits in soybean using SNP markers and SNP-Based haplotype analysis. *PLoS One* 12:e0171105. 10.1371/journal.pone.0171105 28152092 PMC5289539

[B17] Cui Y. Zhang F. Zhou Y. (2018). The application of multi-locus GWAS for the detection of salt-tolerance loci in rice. *Front. Plant Sci.* 9:1464. 10.3389/fpls.2018.01464 30337936 PMC6180169

[B18] Danecek P. Auton A. Abecasis G. Albers C. A. Banks E. DePristo M. A. (2011). The variant call format and VCFtools. *Bioinformatics* 27 2156–2158. 10.1093/bioinformatics/btr330 21653522 PMC3137218

[B19] Delfini J. Moda-Cirino V. dos Santos Neto J. Ruas P. M. Sant’Ana G. C. Gepts P. (2021a). Population structure, genetic diversity and genomic selection signatures among a Brazilian common bean germplasm. *Sci. Rep.* 11:2964. 10.1038/s41598-021-82437-4 33536468 PMC7859210

[B20] Delfini J. Moda-Cirino V. dos Santos Neto J. Zeffa D. M. Nogueira A. F. Ribeiro L. A. B. (2021b). Genome-wide association study for grain mineral content in a Brazilian common bean diversity panel. *Theor. Appl. Genet* 134 2795–2811. 10.1007/s00122-021-03859-2 34027567

[B21] Delfini J. Moda-Cirino V. Ruas C. D. F. Dos Santos Neto J. Ruas P. M. Buratto J. S. (2017). Distinctness of Brazilian common bean cultivars with carioca and black grain by means of morphoagronomic and molecular descriptors. *PLoS One* 12:e0188798. 10.1371/journal.pone.0188798 29190665 PMC5708700

[B22] Falconer D. S. Mackay T. F. C. (1996). *Introduction to Quantitative Genetics.* Essex: Longmam.

[B23] Fang Y. Liu S. Dong Q. Zhang K. Tian Z. Li X. (2020). Linkage analysis and multi-locus genome-wide association studies identify QTNs controlling soybean plant height. *Front. Plant Sci.* 11:9. 10.3389/fpls.2020.00009 32117360 PMC7033546

[B24] Fattahi F. Fakheri B. A. (2019). Evolutionary dynamics models in biometrical genetics supports QTL × environment interactions. *J. Genet.* 98:39. 10.1007/s12041-019-1089-y 31204724

[B25] Gepts P. Kmiecik K. Pereira P. Bliss F. A. (1988). Dissemination pathways of common bean (*Phaseolus vulgaris, Fabaceae*) deduced from phaseolin electrophoretic variability. I. *Am. Econ. Bot.* 42 73–85. 10.1007/BF02859036

[B26] Gupta N. Zargar S. M. Singh R. Nazir M. Mahajan R. Salgotra R. K. (2020). Marker association study of yield attributing traits in common bean (*Phaseolus vulgaris L*.). *Mol. Biol. Rep.* 47 6769–6783. 10.1007/s11033-020-05735-6 32852680

[B27] Hallauer A. R. Miranda Filho J. B. (1988). *Quantitative Genetics in Maize Breeding.* Ames, IA: Iowa State University Press.

[B28] He L. Xiao J. Rashid K. Y. Yao Z. Li P. Jia G. (2019b). Genome-wide association studies for pasmo resistance in flax (*Linum usitatissimum L*.). *Front. Plant Sci.* 9:1982. 10.3389/fpls.2018.01982 30693010 PMC6339956

[B29] He L. Xiao J. Rashid K. Y. Jia G. Li P. Yao Z. (2019a). Evaluation of genomic prediction for Pasmo resistance in flax. *Int. J. Mol. Sci.* 20:359. 10.3390/ijms20020359 30654497 PMC6359301

[B30] Hoyos-Villegas V. Song Q. Kelly J. D. (2017). Genome-wide association analysis for drought tolerance and associated traits in common bean. *Plant Genome* 10 1–17. 10.3835/plantgenome2015.12.0122 28464065

[B31] Ikram M. Han X. Zuo J.-F. Song J. Han C.-Y. Zhang Y.-W. (2020). Identification of QTNs and their candidate genes for 100-seed weight in soybean (*Glycine max L*.) using multi-locus genome-wide association studies. *Genes* 11:714. 10.3390/genes11070714 32604988 PMC7397327

[B32] Kamfwa K. Cichy K. A. Kelly J. D. (2015). Genome-wide association study of agronomic traits in common bean. *Plant Genome* 8 1–12. 10.3835/plantgenome2014.09.0059 33228312

[B33] Keller B. Ariza-Suarez D. de la Hoz J. Aparicio J. S. Portilla-Benavides A. E. Buendia H. F. (2020). Genomic prediction of agronomic traits in common bean (*Phaseolus vulgaris L*.) under environmental stress. *Front. Plant Sci.* 11:1001. 10.3389/fpls.2020.01001 32774338 PMC7381332

[B34] Korte A. Ashley F. (2013). The advantages and limitations of trait analysis with GWAS?: a review self-fertilisation makes *Arabidopsis* particularly well suited to GWAS. *Plant Methods* 9:29. 23876160 10.1186/1746-4811-9-29 PMC3750305

[B35] Kwak M. Gepts P. (2009). Structure of genetic diversity in the two major gene pools of common bean (*Phaseolus vulgaris L., Fabaceae*). *Theor. Appl. Genet.* 118 979–992. 10.1007/s00122-008-0955-4 19130029

[B36] Lei L. Wang L. Wang S. Wu J. (2020). Marker-trait association analysis of seed traits in accessions of common bean (*Phaseolus vulgaris L*.) in China. *Front. Genet.* 11:698. 10.3389/fgene.2020.00698 32714377 PMC7344293

[B37] Li C. Fu Y. Sun R. Wang Y. Wang Q. (2018). Single-locus and multi-locus genome-wide association studies in the genetic dissection of fiber quality traits in upland cotton (*Gossypium hirsutum l*.). *Front. Plant Sci.* 9:1083. 10.3389/fpls.2018.01083 30177935 PMC6109694

[B38] Ma L. Liu M. Yan Y. Qing C. Zhang X. Zhang Y. (2018). Genetic dissection of maize embryonic callus regenerative capacity using multi-locus genome-wide association studies. *Front. Plant Sci.* 9:561. 10.3389/fpls.2018.00561 29755499 PMC5933171

[B39] MacQueen A. H. White J. W. Lee R. Osorno J. M. Schmutz J. Miklas P. N. (2020). Genetic associations in four decades of multienvironment trials reveal agronomic trait evolution in common bean. *Genetics* 215 267–284. 10.1534/genetics.120.303038 32205398 PMC7198278

[B40] Misra G. Badoni S. Domingo C. J. Cuevas R. P. O. Llorente C. Mbanjo E. G. N. (2018). Deciphering the genetic architecture of cooked rice texture. *Front. Plant Sci.* 9:1405. 10.3389/fpls.2018.01405 30333842 PMC6176215

[B41] Moghaddam S. M. Mamidi S. Osorno J. M. Lee R. Brick M. Kelly J. (2016). Genome-wide association study identifies candidate loci underlying agronomic traits in a middle american diversity panel of common bean. *Plant Genome* 9 1–21. 10.3835/plantgenome2016.02.0012 27902795

[B42] Nadeem M. A. Karaköy T. Yeken M. Z. Habyarimana E. Hatipoglu R. Çiftçi V. (2020). Phenotypic characterization of 183 Turkish common bean accessions for agronomic, trading, and consumer-preferred plant characteristics for breeding purposes. *Agronomy* 10:272. 10.3390/agronomy10020272

[B43] Nadeem M. A. Nawaz M. A. Shahid M. Q. Doðan Y. Comertpay G. Yıldız M. (2018). DNA molecular markers in plant breeding: current status and recent advancements in genomic selection and genome editing. *Biotechnol. Biotechnol. Equip.* 32 261–285. 10.1080/13102818.2017.1400401

[B44] Nakano Y. Kobayashi Y. (2020). Genome-wide association studies of agronomic traits consisting of field-and molecular-based phenotypes. *Rev. Agric. Sci.* 8 28–45. 10.7831/ras.8.0_28

[B45] Nascimento M. Nascimento A. C. C. Silva F. F. E. Barili L. D. Do Vale N. M. Carneiro J. E. (2018). Quantile regression for genome-wide association study of flowering time-related traits in common bean. *PLoS One* 13:e0190303. 10.1371/journal.pone.0190303 29300788 PMC5754186

[B46] Nemli S. Asciogul T. K. Kaya H. B. Kahraman A. Eşiyok D. Tanyolac B. (2014). Association mapping for five agronomic traits in the common bean (*Phaseolus vulgaris L*.). *J. Sci. Food Agric.* 94 3141–3151. 10.1002/jsfa.6664 24659306

[B47] Oladosu Y. Rafii M. Y. Samuel C. Fatai A. Magaji U. Kareem I. (2019). Drought resistance in rice from conventional to molecular breeding: a review. *Int. J. Mol. Sci.* 20:3519. 10.3390/ijms20143519 31323764 PMC6678081

[B48] Oladzad A. Porch T. Rosas J. C. Moghaddam S. M. Beaver J. Beebe S. E. (2019). Single and multi-trait GWAS identify genetic factors associated with production traits in common bean under abiotic stress environments. *G3* 9 1881–1892. 10.1534/g3.119.400072 31167806 PMC6553540

[B49] Pan L. He J. Zhao T. Xing G. Wang Y. Yu D. (2018). Efficient QTL detection of flowering date in a soybean RIL population using the novel restricted two-stage multi-locus GWAS procedure. *Theor. Appl. Genet.* 131 2581–2599. 10.1007/s00122-018-3174-7 30167759

[B50] Parker T. A. Berny Mier y Teran J. C. Palkovic A. Jernstedt J. Gepts P. (2020a). Pod indehiscence is a domestication and aridity resilience trait in common bean. *New Phytol.* 225 558–570. 10.1111/nph.16164 31486530

[B51] Parker T. A. Palkovic A. Gepts P. (2020b). Determining the genetic control of common bean early-growth rate using unmanned aerial vehicles. *Remote Sens.* 12:1748. 10.20944/preprints202004.0309.v1 32283112

[B52] Pritchard J. K. Stephens M. Donnelly P. (2000). Inference of population structure using multilocus genotype data. *Genetics* 155 945–959. 10835412 10.1093/genetics/155.2.945 PMC1461096

[B53] Rana J. C. Sharma T. R. Tyagi R. K. Chahota R. K. Gautam N. K. Singh M. (2015). Characterisation of 4274 accessions of common bean (*Phaseolus vulgaris L*.) germplasm conserved in the indian gene bank for phenological, morphological and agricultural traits. *Euphytica* 205 441–457. 10.1007/s10681-015-1406-3

[B54] Rawal V. Navarro D. K. (2019). *The Global Economy of Pulses.* Rome: Food and Agriculture Organization.

[B55] Resende R. T. de Resende M. D. V. Azevedo C. F. Silva F. F. E. Melo L. C. Pereira H. S. (2018). Genome-wide association and regional heritability mapping of plant architecture, lodging and productivity in phaseolus vulgaris. *G3* 8 2841–2854. 10.1534/g3.118.200493 29967054 PMC6071601

[B56] Saltz J. B. Hessel F. C. Kelly M. W. (2017). Trait correlations in the genomics era. *Trends Ecol. Evol.* 32 279–290. 10.1016/j.tree.2016.12.008 28139251

[B57] SAS Institute (2000). *JMP IN 4.0.3.* Cary, NC: SAS Institute.

[B58] Singh S. P. Nodari R. Gepts P. (1991). Genetic diversity in cultivated common bean: i. allozymes. *Crop Sci.* 31:19. 10.2135/cropsci1991.0011183x003100010004x

[B59] Soltani A. Bello M. Mndolwa E. Schroder S. Moghaddam S. M. Osorno J. M. (2016). Targeted analysis of dry bean growth habit: interrelationship among architectural, phenological, and yield components. *Crop Sci.* 56 3005–3015. 10.2135/cropsci2016.02.0119

[B60] Soltani A. MafiMoghaddam S. Walter K. Restrepo-Montoya D. Mamidi S. Schroder S. (2017). Genetic architecture of flooding tolerance in the dry bean middle-American diversity panel. *Front. Plant Sci.* 8:1183. 10.3389/fpls.2017.01183 28729876 PMC5498472

[B61] Tamba C. L. Ni Y. L. Zhang Y. M. (2017). Iterative sure independence screening EM-Bayesian LASSO algorithm for multi-locus genome-wide association studies. *PLoS Comput. Biol.* 13:e1005357. 10.1371/journal.pcbi.1005357 28141824 PMC5308866

[B62] Tamba C. L. Zhang Y.-M. (2018). A fast mrMLM algorithm for multi-locus genome-wide association studies. *Biorxiv* [Preprint] 10.1101/341784 bioRxiv: 341784,

[B63] Teixeira F. F. Ramalho M. A. P. Abreu ÂD. F. B. (1999). Genetic control of plant architecture in the common bean (*Phaseolus vulgaris L*.). *Genet. Mol. Biol.* 22 577–582. 10.1590/S1415-47571999000400019

[B64] Tock A. J. Fourie D. Walley P. G. Holub E. B. Soler A. Cichy K. A. (2017). Genome-wide linkage and association mapping of halo blight resistance in common bean to race 6 of the globally important bacterial pathogen. *Front. Plant Sci.* 8:1170. 10.3389/fpls.2017.01170 28736566 PMC5500643

[B65] Valdisser P. A. Müller B. S. de Almeida Filho J. E. Júnior O. P. M. Guimarães C. M. Borba T. C. (2020). Genome-wide association studies detect multiple QTLs for productivity in Mesoamerican diversity panel of common bean under drought stress. *Front. Plant Sci.* 11:574674. 10.3389/fpls.2020.574674 33343591 PMC7738703

[B66] Wang M. Jiang N. Jia T. Leach L. Cockram J. Waugh R. (2012). Genome-wide association mapping of agronomic and morphologic traits in highly structured populations of barley cultivars. *Theor. Appl. Genet.* 124 233–246. 10.1007/s00122-011-1697-2 21915710

[B67] Wang S. B. Feng J. Y. Ren W. L. Huang B. Zhou L. Wen Y. J. (2016). Improving power and accuracy of genome-wide association studies via a multi-locus mixed linear model methodology. *Sci. Rep.* 6:19444. 10.1038/srep19444 26787347 PMC4726296

[B68] Ward B. P. Brown-Guedira G. Kolb F. L. Van Sanford D. A. Tyagi P. Sneller C. H. (2019). Genome-wide association studies for yield-related traits in soft red winter wheat grown in Virginia. *PLoS One* 14:e0208217. 10.1371/journal.pone.0208217 30794545 PMC6386437

[B69] Wei T. Simko V. (2017). *R Package “corrplot” Visualization of a Correlation Matrix (Version 0.84).*

[B70] Wen Y. J. Zhang H. Ni Y. L. Huang B. Zhang J. Feng J. Y. (2018). Methodological implementation of mixed linear models in multi-locus genome-wide association studies. *Brief. Bioinform.* 19 700–712. 10.1093/bib/bbw145 28158525 PMC6054291

[B71] Wu J. Wang L. Fu J. Chen J. Wei S. Zhang S. (2020). Resequencing of 683 common bean genotypes identifies yield component trait associations across a north–south cline. *Nat. Genet.* 52 118–125. 10.1038/s41588-019-0546-0 31873299

[B72] Yang Y. Chai Y. Zhang X. Lu S. Zhao Z. Wei D. (2020). Multi-locus GWAS of quality traits in bread wheat: mining more candidate genes and possible regulatory network. *Front. Plant Sci.* 11:1091. 10.3389/fpls.2020.01091 32849679 PMC7411135

[B73] Ya-Wen Z. Pei L. Yuan-Ming Z. (2019). *mrMLM.GUI: Multi-Locus Random-SNP-Effect Mixed Linear Model Tools for Genome-Wide Association Study with Graphical User Interface. R Package Version 4.0.*

[B74] Zhang J. Feng J. Y. Ni Y. L. Wen Y. J. Niu Y. Tamba C. L. (2017). PLARmEB: Integration of least angle regression with empirical Bayes for multilocus genome-wide association studies. *Heredity* 118 517–524. 10.1038/hdy.2017.8 28295030 PMC5436030

[B75] Zhang J. Song Q. Cregan P. B. Nelson R. L. Wang X. Wu J. (2015). Genome-wide association study for flowering time, maturity dates and plant height in early maturing soybean (Glycine max) germplasm. *BMC Genomics* 16:217. 10.1186/s12864-015-1441-4 25887991 PMC4449526

[B76] Zhang K. Fan G. Zhang X. Zhao F. Wei W. Du G. (2017). Identification of QTLs for 14 agronomically important traits in Setaria italica based on SNPs generated from high-throughput sequencing. *G3* 7 1587–1594. 10.1534/g3.117.041517 28364039 PMC5427501

[B77] Zhang Y. Liu P. Zhang X. Zheng Q. Chen M. Ge F. (2018). Multi-locus genome-wide association study reveals the genetic architecture of stalk lodging resistance-related traits in maize. *Front. Plant Sci.* 9:611. 10.3389/fpls.2018.00611 29868068 PMC5949362

[B78] Zhang Y. M. Jia Z. Dunwell J. M. (2019). Editorial: the applications of new multi-locus gwas methodologies in the genetic dissection of complex traits. *Front. Plant Sci.* 10:100. 10.3389/fpls.2019.00100 30804969 PMC6378272

