# Supplementary material for: Genome-Wide Association Study Identifies Genomic Regions for Important Morpho-Agronomic Traits in Mesoamerican Common Bean
Source: Front Plant Sci. 2021 Oct 7;12:748829. doi: 10.3389/fpls.2021.748829 (PMC8528967; doi:10.3389/fpls.2021.748829)
Supplement: Supplementary file 1 [file Table_1.DOCX]

Supplementary Material

**Supplementary Table 1 -** QTNs associated with morpho-agronomic traits detected at least twice via different methods and in different environments in common bean accessions belonging to the Brazilian Diversity Panel (BDP).

| **Trait.** | **SNP** | | | **Chr** | | | **Position (bp)** | | | **QTN Effect^1^** | | | **LOD score^2^** | | | **PVE (%)^3^** | | | **MAF^4^** | | | **Gen** | | | **Env^5^** | | | **Methods^6^** | |
| --- | --- | --- | --- | --- | --- | --- | --- | --- | --- | --- | --- | --- | --- | --- | --- | --- | --- | --- | --- | --- | --- | --- | --- | --- | --- | --- | --- | --- | --- |
| PH | S01_5305887 | | | 1 | | | 5305887 | | | -8.13 ~ -6.83 | | | 4.78 ~ 6.56 | | | 6.55 ~ 9.33 | | | 0.063 | | | CC | | | 1 | | | 1,2,3 | |
|  | S02_33714137 | | | 2 | | | 33714137 | | | 1.91 ~ 3.34 | | | 3.41 ~ 4.98 | | | 1.32 ~ 5.36 | | | 0.253 | | | AA | | | 3,4 | | | 1,4 | |
|  | S03_13423968 | | | 3 | | | 13423968 | | | -3.18 ~ -2.99 | | | 3.98 ~ 4.45 | | | 6.17 ~ 6.97 | | | 0.180 | | | GG | | | 5 | | | 1,2 | |
|  | S03_40723746 | | | 3 | | | 40723746 | | | 2.49 ~ 3.09 | | | 3.61 ~ 4.74 | | | 3.43 ~ 5.29 | | | 0.348 | | | CC | | | 1 | | | 1,2 | |
|  | S04_10370006 | | | 4 | | | 10370006 | | | 2.38 ~ 4.53 | | | 3 ~ 4.18 | | | 2.35 ~ 11.84 | | | 0.449 | | | CC | | | 3 | | | 3,4 | |
|  | S04_2493278 | | | 4 | | | 2493278 | | | 2.93 ~ 5.28 | | | 3.71 ~ 3.87 | | | 4.66 ~ 7.89 | | | 0.136 | | | CC | | | 1,5 | | | 1,3 | |
|  | S04_2493297 | | | 4 | | | 2493297 | | | 2.59 ~ 3.9 | | | 3.49 ~ 5.09 | | | 2.2 ~ 4.18 | | | 0.152 | | | GG | | | 1,5 | | | 2,4 | |
|  | S04_373114 | | | 4 | | | 373114 | | | -3.34 ~ -1.73 | | | 3.84 ~ 5.66 | | | 1.4 ~ 4.6 | | | 0.433 | | | GG | | | 3,5 | | | 1,4 | |
|  | S05_39604389 | | | 5 | | | 39604389 | | | 1.65 ~ 3.76 | | | 3.04 ~ 6.12 | | | 2.9 ~ 8.19 | | | 0.455 | | | GG | | | 1,2,3,5 | | | 1,2,3,4 | |
|  | S05_39624676 | | | 5 | | | 39624676 | | | 3.19 ~ 3.44 | | | 7.87 ~ 10.98 | | | 11.72 ~ 13.67 | | | 0.449 | | | GG | | | 5 | | | 1,2 | |
|  | S05_39680093 | | | 5 | | | 39680093 | | | 3.16 ~ 4.28 | | | 4.86 ~ 7.85 | | | 5.51 ~ 10.08 | | | 0.352 | | | GG | | | 1 | | | 1,2,3 | |
|  | S06_25397668 | | | 6 | | | 25397668 | | | 2.43 ~ 2.81 | | | 3.43 ~ 5.09 | | | 2.57 ~ 8.4 | | | 0.354 | | | CC | | | 5 | | | 1,3,4 | |
|  | S06_25791997 | | | 6 | | | 25791997 | | | -7.54 ~ -5.18 | | | 4.73 ~ 8.19 | | | 6.8 ~ 12.64 | | | 0.118 | | | TT | | | 1 | | | 2,4 | |
|  | S07_17942068 | | | 7 | | | 17942068 | | | 5.59 ~ 7.5 | | | 5.24 ~ 5.92 | | | 2.85 ~ 6.82 | | | 0.051 | | | CC | | | 3,4,5 | | | 1,4 | |
|  | S08_3952225 | | | 8 | | | 3952225 | | | -2.74 ~ -2.7 | | | 3.15 ~ 3.87 | | | 4.6 ~ 4.72 | | | 0.455 | | | TT | | | 3 | | | 1,2 | |
|  | S08_58030052 | | | 8 | | | 58030052 | | | 2.67 ~ 5.09 | | | 3.06 ~ 4.19 | | | 2.25 ~ 7.55 | | | 0.148 | | | AA | | | 3 | | | 1,3 | |
|  | S08_62021856 | | | 8 | | | 62021856 | | | 4.08 ~ 8.43 | | | 4.52 ~ 6.2 | | | 1.84 ~ 9.62 | | | 0.062 | | | GG | | | 3,5 | | | 3,4 | |
|  | S10_42297372 | | | 10 | | | 42297372 | | | 3.35 ~ 3.7 | | | 3.83 ~ 4.73 | | | 5 ~ 6.08 | | | 0.244 | | | AA | | | 1 | | | 1,3 | |
|  | S10_43645690 | | | 10 | | | 43645690 | | | 2.16 ~ 4.45 | | | 4.17 ~ 6.92 | | | 3.97 ~ 8.46 | | | 0.242 | | | CC | | | 3,5 | | | 1,2,3,4 | |
|  | S10_44036828 | | | 10 | | | 44036828 | | | -2.83 ~ -1.69 | | | 3.04 ~ 4.97 | | | 3.89 ~ 10.61 | | | 0.393 | | | TT | | | 2 | | | 2,3,4 | |
|  | S11_1465100 | | | 11 | | | 1465100 | | | 2.69 ~ 3.06 | | | 4.78 ~ 5.57 | | | 9.01 ~ 11.3 | | | 0.318 | | | AA | | | 2 | | | 1,2,3 | |
| FPIH | S01_1292307 | | | 1 | | | 1292307 | | | 0.55 ~ 0.59 | | | 3.35 ~ 3.43 | | | 4.77 ~ 5.28 | | | 0.1761 | | | AA | | | 5 | | | 1,3,4 | |
|  | S01_20991636 | | | 1 | | | 20991636 | | | -0.46 ~ -0.37 | | | 3.33 ~ 3.8 | | | 3.62 ~ 5.53 | | | 0.4375 | | | CC | | | 5 | | | 2,3,4 | |
|  | S01_2896512 | | | 1 | | | 2896512 | | | -0.7 ~ -0.67 | | | 3.16 ~ 3.66 | | | 4.21 ~ 4.66 | | | 0.092 | | | AA | | | 5 | | | 1,3 | |
|  | S01_48972163 | | | 1 | | | 48972163 | | | 0.55 ~ 0.63 | | | 4.04 ~ 4.69 | | | 5.3 ~ 6.89 | | | 0.2069 | | | AA | | | 5 | | | 1,3 | |
|  | S03_4648326 | | | 3 | | | 4648326 | | | 0.4 ~ 0.6 | | | 3.39 ~ 3.43 | | | 3.1 ~ 6.69 | | | 0.2159 | | | TT | | | 2 | | | 2,3 | |
|  | S05_39601221 | | | 5 | | | 39601221 | | | 0.4 ~ 0.54 | | | 3.55 ~ 3.95 | | | 3 ~ 5.5 | | | 0.2241 | | | GG | | | 5 | | | 2,3 | |
|  | S06_20814429 | | | 6 | | | 20814429 | | | -0.61 ~ -0.49 | | | 3.84 ~ 6.86 | | | 6.52 ~ 10.05 | | | 0.483 | | | AA | | | 5 | | | 1,2,4 | |
|  | S06_29875948 | | | 6 | | | 29875948 | | | -0.79 ~ -0.55 | | | 3.72 ~ 3.76 | | | 3.72 ~ 7.43 | | | 0.125 | | | CC | | | 2 | | | 2,3 | |
|  | S09_27171634 | | | 9 | | | 27171634 | | | 0.51 ~ 0.78 | | | 3.09 ~ 3.63 | | | 3.03 ~ 6.93 | | | 0.118 | | | CC | | | 2 | | | 2,3,4 | |
|  | S10_15424493 | | | 10 | | | 15424493 | | | 0.45 ~ 0.72 | | | 3.05 ~ 3.35 | | | 2.71 ~ 6.78 | | | 0.1307 | | | AA | | | 2 | | | 2,3 | |
| NN | S01_5448199 | | | 1 | | | 5448199 | | | 0.14 ~ 0.17 | | | 3.33 ~ 4.96 | | | 2.67 ~ 4.24 | | | 0.2584 | | | CC | | | 5 | | | 1,2,3 | |
|  | S02_25464609 | | | 2 | | | 25464609 | | | 0.26 ~ 0.36 | | | 4 ~ 4.71 | | | 4.59 ~ 8.52 | | | 0.4148 | | | GG | | | 1 | | | 2,3,4 | |
|  | S02_25870688 | | | 2 | | | 25870688 | | | 0.35 ~ 0.36 | | | 3.41 ~ 3.97 | | | 3.64 ~ 3.84 | | | 0.1517 | | | GG | | | 4 | | | 2,4 | |
|  | S02_38214788 | | | 2 | | | 38214788 | | | -0.27 ~ -0.25 | | | 4.55 ~ 5.7 | | | 4.9 ~ 5.52 | | | 0.264 | | | GG | | | 2 | | | 2,4 | |
|  | | S02_48537121 | | | 2 | | | 48537121 | | | 0.17 ~ 0.26 | | | 3.03 ~ 3.67 | | | 2.4 ~ 5.55 | | | 0.2898 | | | CC | | | 2 | | 1,3,4 | |
|  | | S04_2503984 | | | 4 | | | 2503984 | | | 0.13 ~ 0.29 | | | 3.67 ~ 5.43 | | | 3.03 ~ 7.81 | | | 0.3807 | | | CC | | | 2,5 | | 1,2,3 | |
|  | | S07_3943786 | | | 7 | | | 3943786 | | | -0.14 ~ -0.14 | | | 3.53 ~ 4.08 | | | 3.46 ~ 3.65 | | | 0.4663 | | | AA | | | 5 | | 1,2 | |
|  | | S07_474203 | | | 7 | | | 474203 | | | 0.12 ~ 0.19 | | | 3.12 ~ 4.81 | | | 2.07 ~ 5 | | | 0.2841 | | | TT | | | 5 | | 1,3,4 | |
|  | | S07_495888 | | | 7 | | | 495888 | | | -0.35 ~ 0 | | | 3.61 ~ 6.22 | | | 0 ~ 10.76 | | | 0.3523 | | | GG | | | 2 | | 1,2,3,4 | |
|  | | S08_44008378 | | | 8 | | | 44008378 | | | -0.47 ~ -0.32 | | | 3.84 ~ 5.58 | | | 3.42 ~ 7.1 | | | 0.0909 | | | CC | | | 2 | | 1,2,3,4 | |
|  | | S08_61614494 | | | 8 | | | 61614494 | | | 0.15 ~ 0.25 | | | 3.93 ~ 7.72 | | | 4.14 ~ 11.33 | | | 0.4045 | | | TT | | | 5 | | 1,2,3 | |
|  | | S09_22575298 | | | 9 | | | 22575298 | | | -0.4 ~ -0.23 | | | 3.53 ~ 4.47 | | | 2.38 ~ 7.16 | | | 0.1348 | | | CC | | | 2 | | 1,3 | |
|  | | S09_5349386 | | | 9 | | | 5349386 | | | 0.25 ~ 0.3 | | | 3.07 ~ 5.28 | | | 2.73 ~ 7.29 | | | 0.125 | | | GG | | | 5 | | 1,2,3,4 | |
|  | | S10_415221 | | | 10 | | | 415221 | | | 0.3 ~ 0.37 | | | 4.18 ~ 5.1 | | | 7.6 ~ 11.3 | | | 0.427 | | | GG | | | 3 | | 1,3 | |
|  | | S10_44010107 | | | 10 | | | 44010107 | | | -0.26 ~ -0.23 | | | 3.48 ~ 4.48 | | | 4.79 ~ 5.96 | | | 0.3708 | | | CC | | | 2 | | 1,2,3 | |
|  | | S11_8369504 | | | 11 | | | 8369504 | | | -0.19 ~ -0.14 | | | 3.3 ~ 4.87 | | | 2.9 ~ 5.43 | | | 0.264 | | | AA | | | 5 | | 1,2,3 | |
| PL | | S02_1040748 | | | 2 | | | 1040748 | | | -0.26 ~ -0.19 | | | 3.43 ~ 4.19 | | | 1.02 ~ 4.9 | | | 0.1023 | | | TT | | | 2 | | 1,3,4 | |
|  | | S02_47586597 | | | 2 | | | 47586597 | | | -0.33 ~ -0.22 | | | 4.92 ~ 8.61 | | | 1.63 ~ 10.93 | | | 0.1685 | | | AA | | | 2,3,5 | | 1,2,3,4 | |
|  | | S02_49444827 | | | 2 | | | 49444827 | | | 0.16 ~ 0.19 | | | 3.29 ~ 4.1 | | | 1.98 ~ 6.66 | | | 0.4943 | | | AA | | | 2 | | 3,4 | |
|  | | S02_49538733 | | | 2 | | | 49538733 | | | 0.13 ~ 0.23 | | | 3.1 ~ 5.31 | | | 2.9 ~ 8.78 | | | 0.5 | | | GG | | | 1,2,3 | | 1,2,3 | |
|  | | S03_1420507 | | | 3 | | | 1420507 | | | -0.14 ~ -0.13 | | | 4.5 ~ 5.06 | | | 1.05 ~ 4.11 | | | 0.4124 | | | TT | | | 5 | | 2,4 | |
|  | | S04_1926044 | | | 4 | | | 1926044 | | | -0.44 ~ -0.34 | | | 3.83 ~ 3.86 | | | 2.45 ~ 6.72 | | | 0.0568 | | | CC | | | 3 | | 3,4 | |
|  | | S07_30515591 | | | 7 | | | 30515591 | | | -0.31 ~ -0.23 | | | 3 ~ 3.65 | | | 3.91 ~ 7.18 | | | 0.1292 | | | GG | | | 3 | | 1,2,3 | |
|  | | S07_30758337 | | | 7 | | | 30758337 | | | -0.31 ~ -0.3 | | | 4.22 ~ 4.84 | | | 5.71 ~ 6.25 | | | 0.0955 | | | CC | | | 2 | | 1,3 | |
|  | | S08_1159693 | | | 8 | | | 1159693 | | | -0.23 ~ -0.19 | | | 3.48 ~ 4.51 | | | 1.52 ~ 5.23 | | | 0.1591 | | | CC | | | 2 | | 1,3,4 | |
|  | | S08_26923941 | | | 8 | | | 26923941 | | | 0 ~ 0.17 | | | 3.08 ~ 3.24 | | | 0 ~ 0.6 | | | 0.0674 | | | CC | | | 2 | | 1,4 | |
|  | | S08_61609638 | | | 8 | | | 61609638 | | | -0.28 ~ -0.27 | | | 3.09 ~ 4.39 | | | 1.45 ~ 3.66 | | | 0.0674 | | | CC | | | 2 | | 1,4 | |
|  | | **S08_62432046** | | | 8 | | | 62432046 | | | -0.23 ~ -0.16 | | | 4.01 ~ 4.24 | | | 1.08 ~ 5.14 | | | 0.1534 | | | TT | | | 2 | | 1,2,3,4 | |
|  | | **S08_9375624** | | | 8 | | | 9375624 | | | -0.54 ~ -0.41 | | | 5.89 ~ 6.76 | | | 2.25 ~ 13.21 | | | 0.0571 | | | CC | | | 5 | | 2,3,4 | |
|  | | S11_2437959 | | | 11 | | | 2437959 | | | 0.17 ~ 0.18 | | | 3.27 ~ 3.39 | | | 3.5 ~ 3.89 | | | 0.2429 | | | AA | | | 4 | | 1,2,4 | |
|  | | S11_47294839 | | | 11 | | | 47294839 | | | -0.38 ~ -0.24 | | | 3.32 ~ 4.14 | | | 2.87 ~ 7.12 | | | 0.0621 | | | CC | | | 5 | | 2,3 | |
| NPP | | S01_25820006 | | | 1 | | | 25820006 | | | 0.73 ~ 0.78 | | | 3.16 ~ 3.62 | | | 2.84 ~ 3.3 | | | 0.4719 | | | CC | | | 3 | | 2,4 | |
|  | | S03_12044967 | | | 3 | | | 12044967 | | | -2.28 ~ -1.62 | | | 3.28 ~ 4.46 | | | 4.9 ~ 9.69 | | | 0.0966 | | | AA | | | 3 | | 1,2,3,4 | |
|  | | S05_40466290 | | | 5 | | | 40466290 | | | 1.04 ~ 1.41 | | | 3.12 ~ 5.36 | | | 3.96 ~ 7.21 | | | 0.2102 | | | TT | | | 3 | | 1,2,3,4 | |
|  | | S05_445417 | | | 5 | | | 445417 | | | -1.43 ~ -0.92 | | | 3.11 ~ 4.96 | | | 2.55 ~ 6.02 | | | 0.0562 | | | AA | | | 5 | | 1,2,3 | |
|  | | S05_4994208 | | | 5 | | | 4994208 | | | -1.39 ~ -1.22 | | | 3.79 ~ 4.92 | | | 2.05 ~ 6.01 | | | 0.1854 | | | CC | | | 4 | | 2,4 | |
|  | | S07_38456082 | | | 7 | | | 38456082 | | | -1.56 ~ -0.48 | | | 3.11 ~ 3.85 | | | 3.31 ~ 11.71 | | | 0.4432 | | | GG | | | 3,4,5 | | 1,2,3,4 | |
|  | | S08_2493035 | | | 8 | | | 2493035 | | | -2.45 ~ -2.07 | | | 5.78 ~ 6.16 | | | 4.1 ~ 11.68 | | | 0.1067 | | | CC | | | 4 | | 1,2,4 | |
|  | | S08_60292408 | | | 8 | | | 60292408 | | | 1.25 ~ 1.73 | | | 3.45 ~ 4.05 | | | 3.1 ~ 4.26 | | | 0.0506 | | | CC | | | 3,5 | | 1 | |
|  | | **S11_1617681** | | | 11 | | | 1617681 | | | -0.97 ~ -0.73 | | | 4.94 ~ 7.45 | | | 7.5 ~ 13.04 | | | 0.4602 | | | AA | | | 5 | | 1,2,3,4 | |
| LP | | S01_221817 | | | 1 | | | 221817 | | | 0.07 ~ 0.14 | | | 3.3 ~ 5.73 | | | 1.92 ~ 7.72 | | | 0.2921 | | | GG | | | 1 | | 1,2,4 | |
|  | | S01_45434768 | | | 1 | | | 45434768 | | | 0.18 ~ 0.22 | | | 3.47 ~ 4.97 | | | 4.39 ~ 7 | | | 0.0674 | | | TT | | | 3 | | 2,4 | |
|  | | S02_41632778 | | | 2 | | | 41632778 | | | 0 ~ 0.18 | | | 3.03 ~ 3.78 | | | 0 ~ 6.56 | | | 0.0629 | | | TT | | | 5 | | 1,3,4 | |
|  | | S02_42213511 | | | 2 | | | 42213511 | | | -0.13 ~ -0.1 | | | 3.27 ~ 5.53 | | | 2.96 ~ 5.33 | | | 0.1854 | | | AA | | | 1 | | 1,4 | |
|  | | | S04_3837889 | | | 4 | | | 3837889 | | | -0.14 ~ -0.11 | | | 3.8 ~ 4 | | | 5.09 ~ 7.31 | | | 0.3427 | | | GG | | | 2 | | 2,3 |
|  | | | S05_36623179 | | | 5 | | | 36623179 | | | -0.13 ~ -0.09 | | | 3.18 ~ 3.46 | | | 3.58 ~ 6.07 | | | 0.2472 | | | GG | | | 3 | | 1,3 |
|  | | | S07_136162 | | | 7 | | | 136162 | | | 0.12 ~ 0.13 | | | 3.51 ~ 5.46 | | | 5.14 ~ 5.56 | | | 0.2022 | | | AA | | | 3 | | 2,4 |
|  | | | S07_33862545 | | | 7 | | | 33862545 | | | -0.17 ~ -0.1 | | | 3.41 ~ 5.07 | | | 3.24 ~ 9.48 | | | 0.1854 | | | AA | | | 3 | | 1,2,3 |
|  | | | S07_3955474 | | | 7 | | | 3955474 | | | 0.12 ~ 0.14 | | | 3.42 ~ 4.09 | | | 4.84 ~ 6.07 | | | 0.1685 | | | CC | | | 3 | | 2,3 |
|  | | | S07_5382811 | | | 7 | | | 5382811 | | | 0.11 ~ 0.13 | | | 3.67 ~ 4.48 | | | 5.66 ~ 7.92 | | | 0.4607 | | | CC | | | 2 | | 1,3 |
|  | | | **S08_9375624** | | | 8 | | | 9375624 | | | -0.24 ~ -0.15 | | | 3.02 ~ 4.86 | | | 4.08 ~ 9.32 | | | 0.0514 | | | CC | | | 5 | | 1,2,3,4 |
|  | | | S10_41807590 | | | 10 | | | 41807590 | | | 0.12 ~ 0.21 | | | 3.08 ~ 3.61 | | | 2.34 ~ 6.55 | | | 0.0899 | | | GG | | | 2 | | 2,3 |
|  | | | S10_4876917 | | | 10 | | | 4876917 | | | 0.12 ~ 0.14 | | | 3.41 ~ 3.84 | | | 5.38 ~ 6.67 | | | 0.2045 | | | AA | | | 3 | | 2,3,4 |
|  | | | S10_4911729 | | | 10 | | | 4911729 | | | 0.09 ~ 0.12 | | | 3.55 ~ 4.21 | | | 4.86 ~ 8.64 | | | 0.24 | | | GG | | | 5 | | 1,2,3,4 |
|  | | | S11_26987117 | | | 11 | | | 26987117 | | | -0.24 ~ 0 | | | 3.13 ~ 5.86 | | | 0 ~ 5.91 | | | 0.0625 | | | GG | | | 2 | | 3,4 |
|  | | | S11_29062 | | | 11 | | | 29062 | | | -0.13 ~ -0.07 | | | 3.42 ~ 9.02 | | | 3.78 ~ 7.81 | | | 0.3371 | | | TT | | | 1,5 | | 2,3,4 |
|  | | | S11_30320686 | | | 11 | | | 30320686 | | | 0.04 ~ 0.14 | | | 3.09 ~ 3.15 | | | 1.07 ~ 9.14 | | | 0.3933 | | | TT | | | 3 | | 1,3 |
|  | | | S11_52195944 | | | 11 | | | 52195944 | | | -0.1 ~ -0.07 | | | 3.68 ~ 4.5 | | | 4.56 ~ 8.66 | | | 0.3616 | | | GG | | | 5 | | 1,2,3 |
| SP | | | S01_112055 | | | 1 | | | 112055 | | | 0.12 ~ 0.2 | | | 3.97 ~ 4.81 | | | 3.56 ~ 11.2 | | | 0.2727 | | | CC | | | 1 | | 2,3,4 |
|  | | | S01_14589174 | | | 1 | | | 14589174 | | | -0.16 ~ -0.09 | | | 3.28 ~ 4.73 | | | 3.05 ~ 8.67 | | | 0.4382 | | | GG | | | 2 | | 1,3 |
|  | | | S01_465127 | | | 1 | | | 465127 | | | 0.2 ~ 0.28 | | | 3.58 ~ 4.4 | | | 5.07 ~ 9.01 | | | 0.0787 | | | CC | | | 3 | | 2,3 |
|  | | | S05_19626048 | | | 5 | | | 19626048 | | | 0.14 ~ 0.19 | | | 3.93 ~ 4.26 | | | 5.16 ~ 9 | | | 0.191 | | | CC | | | 3 | | 2,3 |
|  | | | S05_36420518 | | | 5 | | | 36420518 | | | 0.1 ~ 0.26 | | | 3.59 ~ 5.91 | | | 2.55 ~ 7.7 | | | 0.0843 | | | GG | | | 4,5 | | 2 |
|  | | | S07_17126884 | | | 7 | | | 17126884 | | | 0.11 ~ 0.19 | | | 3.28 ~ 3.43 | | | 3.3 ~ 3.66 | | | 0.1124 | | | AA | | | 4,5 | | 4 |
|  | | | S08_10350174 | | | 8 | | | 10350174 | | | -0.37 ~ -0.13 | | | 3.27 ~ 4.79 | | | 2.5 ~ 10.42 | | | 0.0568 | | | GG | | | 2,5 | | 1,2,3,4 |
|  | | | S10_42038210 | | | 10 | | | 42038210 | | | 0.08 ~ 0.11 | | | 3.99 ~ 4.5 | | | 5.14 ~ 8.44 | | | 0.3933 | | | GG | | | 5 | | 2,3 |
| TSW | | | S01_44752890 | | | 1 | | | 44752890 | | | 1.98 ~ 2.51 | | | 3.53 ~ 4.97 | | | 4.17 ~ 8.25 | | | 0.1486 | | | CC | | | 4 | | 1,2,3,4 |
|  | | | S02_2242481 | | | 2 | | | 2242481 | | | -1.92 ~ -1.31 | | | 3.4 ~ 6.12 | | | 2.89 ~ 6.49 | | | 0.2147 | | | TT | | | 4 | | 1,2,4 |
|  | | | S03_51482697 | | | 3 | | | 51482697 | | | -0.98 ~ -0.65 | | | 3.04 ~ 4.15 | | | 3.53 ~ 7.5 | | | 0.2429 | | | TT | | | 5 | | 2,3 |
|  | | | S04_41451220 | | | 4 | | | 41451220 | | | -2.03 ~ -1.53 | | | 3.18 ~ 5.58 | | | 5.77 ~ 10.15 | | | 0.1136 | | | CC | | | 1 | | 2,3,4 |
|  | | | S06_640352 | | | 6 | | | 640352 | | | 0 ~ 2.81 | | | 3.75 ~ 4.84 | | | 0 ~ 6.42 | | | 0.0847 | | | AA | | | 4 | | 1,2 |
|  | | | S10_18589262 | | | 10 | | | 18589262 | | | -3.58 ~ -1.56 | | | 3.48 ~ 4.62 | | | 4.49 ~ 8.69 | | | 0.0506 | | | GG | | | 3,5 | | 1,2,3,4 |
|  | | | **S11_1617681** | | | 11 | | | 1617681 | | | -1.21 ~ -0.73 | | | 3.75 ~ 7.98 | | | 5.09 ~ 15.42 | | | 0.4571 | | | AA | | | 5 | | 1,2,3,4 |
|  | | | S11_36831666 | | | 11 | | | 36831666 | | | -1.09 ~ -1 | | | 3.3 ~ 3.3 | | | 4.07 ~ 4.82 | | | 0.2079 | | | AA | | | 1 | | 1,2 |
|  | | | S11_52088416 | | | 11 | | | 52088416 | | | -1.75 ~ -1.48 | | | 4.14 ~ 4.14 | | | 5.38 ~ 7.53 | | | 0.2247 | | | AA | | | 3 | | 1,2,3 |
| W100 | | | S02_2696078 | | | 2 | | | 2696078 | | | 1.15 ~ 1.54 | | | 4.37 ~ 6.73 | | | 5.78 ~ 12.4 | | | 0.0625 | | | GG | | | 1 | | 3,4 |
|  | | | S02_3942810 | | | 2 | | | 3942810 | | | 0.92 ~ 1.15 | | | 3.71 ~ 4.13 | | | 3.75 ~ 5.76 | | | 0.0899 | | | CC | | | 2 | | 2,3 |
|  | | | S03_11484802 | | | 3 | | | 11484802 | | | -0.76 ~ -0.69 | | | 4.42 ~ 5.35 | | | 5.42 ~ 6.48 | | | 0.4034 | | | CC | | | 3 | | 1,2,3,4 |
|  | | | S03_4682037 | | | 3 | | | 4682037 | | | 0.96 ~ 1.68 | | | 3.33 ~ 7.64 | | | 4.04 ~ 12.3 | | | 0.0686 | | | CC | | | 5 | | 2,3,4 |
|  | | | S04_41872476 | | | 4 | | | 41872476 | | | -0.85 ~ -0.76 | | | 3.14 ~ 5.75 | | | 5.43 ~ 6.43 | | | 0.2921 | | | AA | | | 2,4 | | 2,4 |
|  | | | S05_262416 | | | 5 | | | 262416 | | | 0.61 ~ 0.87 | | | 3.23 ~ 4.03 | | | 3.35 ~ 6.97 | | | 0.4802 | | | CC | | | 4 | | 2,3 |
|  | | | S06_1330875 | | | 6 | | | 1330875 | | | 0.57 ~ 0.74 | | | 3.85 ~ 4.04 | | | 3.42 ~ 5.91 | | | 0.1943 | | | TT | | | 5 | | 3,4 |
|  | | | S06_23116097 | | | 6 | | | 23116097 | | | 0.46 ~ 0.87 | | | 3.73 ~ 4.45 | | | 2.77 ~ 9.67 | | | 0.3989 | | | CC | | | 2 | | 2,3 |
|  | | | **S08_62432046** | | | 8 | | | 62432046 | | | -0.85 ~ -0.82 | | | 3.38 ~ 5.44 | | | 4.59 ~ 5.05 | | | 0.1517 | | | TT | | | 2 | | 2,3 |
| YLD | | | S01_1973183 | | | 1 | | | 1973183 | | | 175.24 ~ 289.53 | | | 3.04 ~ 3.23 | | | 1.41 ~ 5.75 | | | 0.0682 | | | GG | | | 3 | | 3,4 |
|  | | | S01_44911599 | | | 1 | | | 44911599 | | | 360.06 ~ 529 | | | 3.93 ~ 6.9 | | | 6.17 ~ 13.31 | | | 0.0966 | | | AA | | | 4 | | 1,2,3,4 |
|  | | | S01_51067135 | | | 1 | | | 51067135 | | | 77.81 ~ 123.27 | | | 3.53 ~ 4.87 | | | 2.65 ~ 6.45 | | | 0.2898 | | | GG | | | 5 | | 1,2,3,4 |
|  | | | S01_6568871 | | | 1 | | | 6568871 | | | -180.61 ~ -173.95 | | | 3.36 ~ 4.29 | | | 6.04 ~ 6.52 | | | 0.2416 | | | CC | | | 3 | | 2,3 |
|  | | | S02_2153254 | | | 2 | | | 2153254 | | | 136.73 ~ 150.71 | | | 3.53 ~ 3.58 | | | 1.89 ~ 3.02 | | | 0.0674 | | | CC | | | 5 | | 2,4 |
|  | | | S02_34513049 | | | 2 | | | 34513049 | | | -186.17 ~ -114.43 | | | 3.33 ~ 4.19 | | | 1.47 ~ 5.84 | | | 0.1875 | | | TT | | | 3 | | 2,3,4 |
|  | | | S02_8413809 | | | 2 | | | 8413809 | | | 167.94 ~ 222.58 | | | 3.87 ~ 3.87 | | | 5.84 ~ 9.95 | | | 0.2191 | | | TT | | | 2 | | 1,3 |
|  | | | S03_2802438 | | | 3 | | | 2802438 | | | 113.41 ~ 141.54 | | | 3.74 ~ 5.64 | | | 6.09 ~ 9.49 | | | 0.3466 | | | CC | | | 1 | |  |
|  | | | S03_3510073 | | | 3 | | | 3510073 | | | 81.7 ~ 126.8 | | | 3.13 ~ 3.92 | | | 3.52 ~ 8.21 | | | 0.4663 | | | CC | | | 5 | | 2,3 |
|  | | | S03_3530662 | | | 3 | | | 3530662 | | | 97.86 ~ 123.61 | | | 3.33 ~ 4.79 | | | 3.82 ~ 4.58 | | | 0.4551 | | | TT | | | 2,5 | | 2,4 |
|  | | | S03_49731981 | | | 3 | | | 49731981 | | | -176.06 ~ -133.48 | | | 4.83 ~ 5.89 | | | 5.24 ~ 11.65 | | | 0.2443 | | | CC | | | 5 | | 1,2,3,4 |
|  | | | S04_47436704 | | | 4 | | | 47436704 | | | 157.01 ~ 180.08 | | | 3.28 ~ 3.89 | | | 4.15 ~ 5.46 | | | 0.191 | | | AA | | | 3 | | 2,3 |
|  | | | S07_34450891 | | | 7 | | | 34450891 | | | 166.38 ~ 260.05 | | | 3.13 ~ 8.89 | | | 3.77 ~ 12.15 | | | 0.1307 | | | AA | | | 2,4,5 | | 1,2,3,4 |
|  | | | S10_2336773 | | | 10 | | | 2336773 | | | -182.4 ~ -146.16 | | | 3.73 ~ 3.73 | | | 5.52 ~ 8.34 | | | 0.309 | | | GG | | | 2 | | 1,3 |
|  | | | S11_43243770 | | | 11 | | | 43243770 | | | 136.79 ~ 170.39 | | | 3.06 ~ 4.47 | | | 3.73 ~ 5.78 | | | 0.2079 | | | CC | | | 2 | | 2,4 |
|  | | | S11_53567276 | | | 11 | | | 53567276 | | | -256.31 ~ -192.42 | | | 3.25 ~ 3.33 | | | 3.29 ~ 5.84 | | | 0.2022 | | | AA | | | 4 | | 2,3 |

PH = plant height (cm), FPIH = first pod insertion height (cm), NN = number of nodules, PL = pod length (cm), NPP = total number of pods per plant, LP = number of locules per pod, SP = number of seeds per pod, TSW = total seed weight per plant (gm), W100 = 100-seed weight (gm) and YLD = grain yield (kg.ha^-1^). ^1^*Quantitative* *trait nucleotide effect;* ^2^LOD value, the significant threshold for *P-value* transformed; ^3^PVE (%): *Phenotypic variation explained*; ^4^*Minor* *allele frequency*; ^5^Environments: 1-LDA, 2-PG, 3-GUA, 4-LSmeans; ^6^Methods: 1-FASTmrMLM, 2-ISIS EM-BLASSE, 3-mrMLM, 4-pLARmEB; Pleiotropic QTNs, related to more than one mineral, are in bold.
